# A Cell-Based Nasal Model for Screening the Deposition, Biocompatibility, and Transport of Aerosolized PLGA Nanoparticles

**DOI:** 10.1021/acs.molpharmaceut.3c00639

**Published:** 2024-02-09

**Authors:** Aida Maaz, Ian S. Blagbrough, Paul A. De Bank

**Affiliations:** ^†^Department of Life Sciences, ^‡^Centre for Therapeutic Innovation, and ^§^Centre for Bioengineering & Biomedical Technologies, University of Bath, Bath BA2 7AY, U.K.

**Keywords:** PLGA nanoparticles, blood–brain barrier, nose-to-brain drug delivery, RPMI 2650, olfactory, air−liquid interface

## Abstract

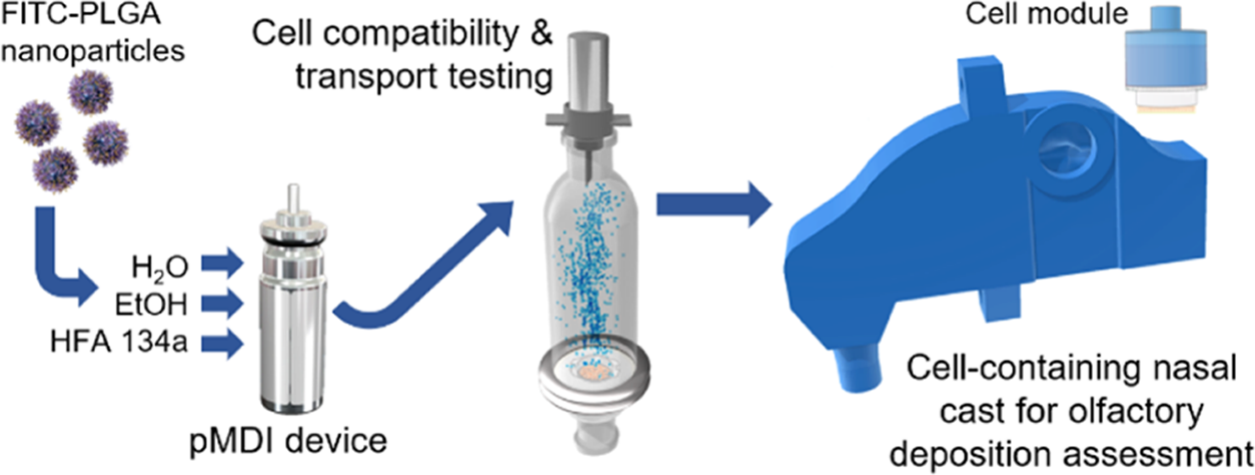

The olfactory region of the nasal cavity directly links the brain to the external environment, presenting a potential direct route to the central nervous system (CNS). However, targeting drugs to the olfactory region is challenging and relies on a combination of drug formulation, delivery device, and administration technique to navigate human nasal anatomy. In addition, in vitro and in vivo models utilized to evaluate the performance of nasal formulations do not accurately reflect deposition and uptake in the human nasal cavity. The current study describes the development of a respirable poly(lactic-*co*-glycolic acid) nanoparticle (PLGA NP) formulation, delivered via a pressurized metered dose inhaler (pMDI), and a cell-containing three-dimensional (3D) human nasal cast model for deposition assessment of nasal formulations in the olfactory region. Fluorescent PLGA NPs (193 ± 3 nm by dynamic light scattering) were successfully formulated in an HFA134a-based pMDI and were collected intact following aerosolization. RPMI 2650 cells, widely employed as a nasal epithelial model, were grown at the air–liquid interface (ALI) for 14 days to develop a suitable barrier function prior to exposure to the aerosolized PLGA NPs in a glass deposition apparatus. Direct aerosol exposure was shown to have little effect on cell viability. Compared to an aqueous NP suspension, the transport rate of the aerosolized NPs across the RPMI 2650 barrier was higher at all time points indicating the potential advantages of delivery via aerosolization and the importance of employing ALI cellular models for testing respirable formulations. The PLGA NPs were then aerosolized into a 3D-printed human nasal cavity model with an insert of ALI RPMI 2650 cells positioned in the olfactory region. Cells remained highly viable, and there was significant deposition of the fluorescent NPs on the ALI cultures. This study is a proof of concept that pMDI delivery of NPs is a viable means of targeting the olfactory region for nose-to-brain drug delivery (NTBDD). The cell-based model allows not only maintenance under ALI culture conditions but also sampling from the basal chamber compartment; hence, this model could be adapted to assess drug deposition, uptake, and transport kinetics in parallel under real-life settings.

## 1 Introduction

Over the last two decades, the nasal route has emerged as an attractive and noninvasive approach for direct drug delivery to the central nervous system (CNS), circumventing the blood–brain barrier (BBB), which is the major obstacle to more effective treatments for CNS diseases and disorders.^1^ The region of the nasal cavity targeted for efficient nose-to-brain drug delivery (NTBDD) is the olfactory epithelium, which accounts for less than 10% of the human nasal cavity and is located in the uppermost region, making it difficult to effectively target. To date, clinical translation of NTBDD remains poorly established due to limitations associated with anatomical and physiological features of the nasal cavity. These include mucociliary clearance, the poor accessibility of the olfactory region, and the complex interplay between formulation-, device-, and patient-related factors in addition to ambiguities in the in vitro–in vivo correlation of NTBDD outcomes.^2^ Nonetheless, the olfactory epithelium remains an attractive drug delivery target and there is significant ongoing research into novel formulations^3^ and devices^4^ to maximize both the deposition in this region and the subsequent delivery of drugs into the CNS.

A number of types of formulations have been utilized for intranasal drug delivery, including solutions, suspensions, emulsions, gels, and powders. For specific targeting of the olfactory epithelium for NTBDD, the formulation must be delivered as small particles or droplets to navigate the narrow nasal valve and avoid impact in the anterior region of the nasal cavity. However, even if localization to the olfactory region is achieved, degradation or clearance of the drug must be avoided for successful absorption across the epithelium. A potential formulation strategy that may overcome some of the hurdles of NTBDD is the use of nanocarriers,^5^ which, depending on the type of particle, have a number of possible advantages over traditional formulations including improved drug stability, prolonged residence time, controlled release, decreased dosing frequency, and facilitated transport. There have been numerous reports of nanocarriers for NTBDD^5^ including liposomes,^6−8^ nanostructured lipid carriers,^9,10^ solid lipid nanocarriers,^11,12^ nanoemulsions,^13,14^ and polymeric nanoparticles. For the latter, a number of polymers have been utilized including natural polymers such as alginate^15^ and chitosan^16^ as well as synthetic polymers such as poly(lactic acid),^17^ polycaprolactone,^18^ and poly(lactic-*co*-glycolic acid) (PLGA).^19^ However, nanoparticle NTBDD studies have been largely confined to liquid formulations, and in vivo experiments have employed animal models that have significant anatomical and physiological differences compared to the human nasal cavity. As a result, flooding of the nasal cavity and deposition of the nanoformulation throughout the epithelium when pipetting into the nostril likely results in rapid clearance and short retention time. Even when delivered as atomized droplets, nanoparticle deposition is controlled by the droplet size in which they are suspended and not by the properties of the nanoparticles themselves. An alternative approach for NTBDD using nanoparticles is the delivery of dry, aerosolized particles to the nasal cavity utilizing a device that contains little to no solvent. As such, their nasal deposition will be mainly influenced by their dimensions and concentration, and, simultaneously, will enable the evaluation of nasal deposition using suitable device and administration techniques, which largely impact the aerosol performance of inhaled formulations in real-life conditions.

The ideal scenario to assess the extent of formulation deposition and drug absorption is the use of advanced imaging techniques^20^ in human subjects in tandem with pharmacokinetic and pharmacodynamic studies. For early-stage research and development, this is clearly unfeasible, so, while human trials are essential for clinical translation, considerable efforts have been made to refine preclinical intranasal drug delivery models.^21^ Mucoadhesion and permeation tests can be performed with ex vivo models, where intact nasal tissue is excised from an animal or human donor, and/or in vitro cultures of primary or immortalized cells such as the RPMI 2650 and Calu-3 cell lines.^22^ Ex vivo models involve critical tissue handling procedures and exhibit species-specific characteristics such as tissue thickness and variations in enzyme expression and activity.^23^ In contrast, cell-based in vitro models are more facile for routine testing and present a reliable, low-cost, and high-throughput evaluation tool in early stages of nasal product development.^24^ The RPMI 2650 cell line, first isolated in 1963 from an anaplastic squamous cell carcinoma of the nasal septum,^25,26^ has become the in vitro model of choice for nasal drug transport and permeation studies. Under air–liquid interface culture conditions, mimicking the physiology of the nasal epithelium, RPMI 2650 cells develop tight junctions, differentiate (developing beating cilia and secreting a mucoid substance), and demonstrate sufficient transepithelial electrical resistance (TEER) values.^27−31^ Recent work has endeavored to develop in vitro models with improved similarity to physiological conditions of the nasal epithelium. These include a nose-on-a-chip system where airflow over RPMI 2650 cells could be tailored to mimic different regions of the nasal cavity,^32^ and a mucosa-on-chip which is capable of monitoring real-time drug transport across an RPMI 2650 epithelial model and has integrated electrodes for TEER measurements.^33^

While in vitro models represent a promising approach for screening transepithelial drug transport under biorelevant conditions, other models are required to assess formulation distribution following nasal delivery, a key consideration for intranasal drug delivery in general and particularly for NTBDD. Most of the research providing a proof of concept for olfactory targeting has utilized pharmacokinetic and pharmacodynamic studies in animal models, mainly rodents. However, as mentioned above, the substantial anatomical and physiological differences between these model animals and the human nasal cavity mean that the results of these in vivo studies are unlikely to translate to the outcomes that would be observed in human subjects.^34^ Furthermore, animal models are usually not suitable for the testing of advanced delivery devices that target the olfactory region. As a result, they are largely limited to the instillation of liquid formulations in the nasal cavity. Attempts have been made to extrapolate in vitro distribution and uptake studies to assess the promise of formulations for human intranasal delivery. For example, Pozzoli et al. cultured RPMI 2650 cells on Snapwell inserts and incorporated them within a three-dimensional (3D) printed expansion chamber attached to a British Pharmacopoeia Apparatus E, Next Generation Impactor. This system was used to determine the deposition and transport of a commercial budesonide formulation and has also been used to examine the suitability of a dry powder formulation for intranasal delivery.^35^ While the combined use of an impactor with an epithelial cell model is a valuable approach to assess the performance and potential of intranasal formulations, for a truly biomimetic system, the complex anatomy of the human nasal cavity should also be considered. One means of achieving this is the use of 3D-printed nasal replicas, which have been a useful tool in bridging the gap between formulation properties, administration device features, and their effect on deposition.^36^ However, nasal casts do not reflect the functional features of the nasal mucosa, such as mucus secretion, or provide information about the transport of the tested formulation across the nasal epithelium.

This paper aims to address current gaps in the formulation of nanocarriers and subsequent in vitro testing of deposition and transport for NTBDD. To improve the penetration of nanoparticles toward the olfactory region, we report the development of a fluorescent PLGA nanoparticle formulation which is delivered from a pressurized metered dose inhaler (pMDI) using a hydrofluoroalkane propellant. The compatibility of this formulation with an RPMI 2650 epithelial model was assessed to determine its suitability for intranasal application and, by incorporating an RPMI 2650 culture in the olfactory region of a 3D-printed human nasal cavity replica, we demonstrate a proof-of-concept system for the simultaneous deposition, biocompatibility, and permeation testing of NTBDD formulations.

## 2 Materials and Methods

### 2.1 Materials

For FITC-PLGA NP synthesis, poly(lactide-*co*-glycolide)-fluorescein (FITC-PLGA; lactide/glycolide 50:50, *M*_W_ 10–20 kDa), poly(1-vinyl-2-pyrrolidone) Kollidon 25 (PVP-K25), and sodium chloride (NaCl) were purchased from Sigma-Aldrich (Germany), and poly(vinyl alcohol) (PVA; 88% hydrolyzed, *M*_W_ 22 kDa) was purchased from Acros Organics (Belgium). For cell culture and cell experiments, RPMI 2650 cells were purchased from ECACC (Cat. No. 88031602; U.K.), Eagle’s minimum essential medium (EMEM) with l-glutamine, heat-inactivated fetal bovine serum (FBS), nonessential amino acids solution (NEAA), penicillin/streptomycin (P/S) antibiotic solution, rat tail collagen type I solution, Hank’s balanced salt solution (HBSS), and LIVE/DEAD cell double staining kit were purchased from Sigma-Aldrich (Germany). Phosphate buffered saline (PBS), 4-(2-hydroxyethyl)–1-piperazine-ethanesulfonic acid (HEPES), and Snapwell (3801) 6-well plate with Polyester (PET) membrane inserts (0.4 μm pore size, 1.12 cm^2^ surface area) were purchased from Thermo Fisher Scientific (U.K.). ThinCert (PET) 12-well culture inserts (0.4 μm pore size, 1.13 cm^2^ surface area) were purchased from Greiner Bio-One (Austria).

### 2.2 Synthesis of FITC-PLGA NPs

Fluorescent PLGA NPs were produced by the nanoprecipitation method. In brief, a 1% (w/v) solution of FITC-PLGA in acetone was added dropwise to an aqueous solution containing the surfactants PVA and PVP-K25 (both 0.5% (w/v)) under continuous magnetic stirring. The colloidal suspension was then rapidly diluted 5-fold in NaCl solution (25 mM) and stirring was continued for 2 h to allow complete removal of the acetone. A detailed protocol is provided in Supporting Information, Section S1.2.

### 2.3 pMDI Formulation

An appropriate mass of lyophilized FITC-PLGA NPs (0.1% w/w of the total pMDI formulation) was resuspended in Milli-Q water (2% w/w) and transferred into 17 mL aluminum canisters. Ethanol (2% w/w) as a cosolvent was subsequently added, and a 50 μL valve was crimped onto the canister using a manual single-unit crimper (Laboratory Plant 02016, Pamasol Willi Mäder AG). The balance of HFA134a propellant was filled through the valve, and the final product was vortexed for 90 s. The canisters were stored inverted for at least 72 h at 20 °C to allow the valve to expand before aerosol performance testing. For consistent conditions, the devices were initially primed 3–5 times to waste in all experiments.

### 2.4 Aerosol Deposition Apparatus

**Figure 1:**
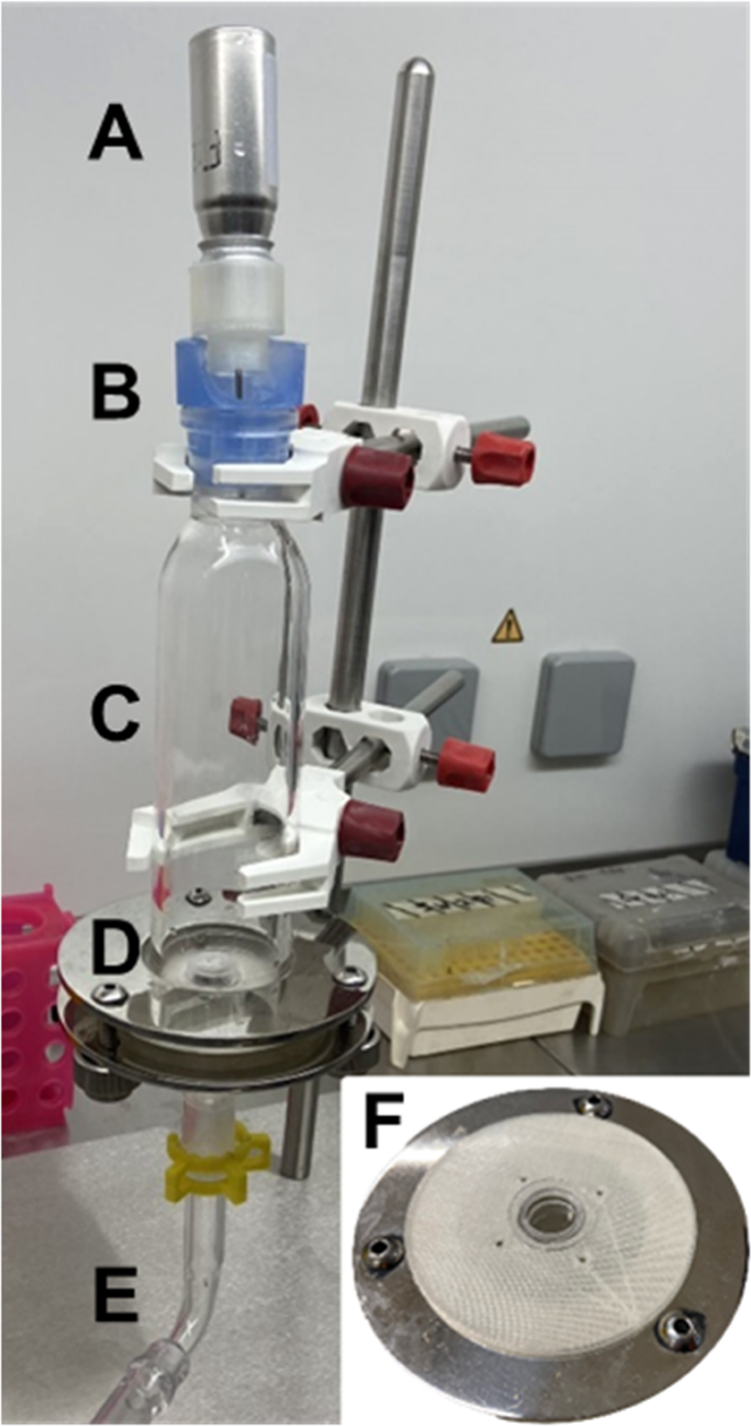
Aerosol deposition apparatus. (A) pMDI cannister at the inlet; (B) rubber adapter; (C) aerosol exposure chamber; (D) support disk to hold filters or a Snapwell holder for nanoparticle collection; (E) outlet to vacuum pump to generate 15 L/min airflow; (F) close-up view of 3D-printed Snapwell holder for cell/aerosol experiments.

An aerosol deposition apparatus was assembled to study: (1) the deposition of aerosolized PLGA NPs on cell-free mixed cellulose esters (MCE) membranes, mica, or ThinCert inserts; (2) the integrity of RPMI 2650 cell layers following aerosol exposure; and (3) the cell permeability of aerosolized NPs. The system (Figure 1) was assembled from a glass Sample Collection Apparatus for FP/Salmeterol Powders (Cat. No. 8640, Copley Scientific, U.K.), commonly used for dosage uniformity analysis of inhaled powders. At the outlet, the apparatus was connected to a rotary vacuum pump (GAST 1423–103Q-G626X) to generate inspiratory flow, which was manually adjusted to a continuous rate of 15 ± 0.2 L/min to simulate moderate human breathing using a calibrated flow meter (DFM 2000, Copley Scientific, U.K.). The aerosolization unit was placed at the inlet and consisted of the pMDI device attached to an in-house printed actuator connected to a needle (i.d.1.6 mm, length 40 mm; Figure 1A,1B). The device was fitted on top of the exposure chamber by using a rubber adaptor at a distance of 150 mm from the target. Aerosols were collected at the distal end of the glass chamber. For NP characterization (Section 2.5), an MEC membrane was clamped between the two sections of the chamber (Figure 1D). NPs were either collected directly onto the membrane or on a piece of mica placed in the center of the membrane. For aerosol deposition and cell exposure experiments, a custom-designed housing (Ø = 69 mm) to accommodate a single ThinCert insert was 3D-printed and clamped between the sections of the chamber (Figure 1F).

### 2.5 Physiochemical Characterization and Quantitative Analysis of FITC-PLGA NPs

The hydrodynamic size and polydispersity index (PDI) of the FITC-PLGA NPs were measured at 20 °C in triplicate by dynamic light scattering using a Zetasizer Nano ZS (Malvern Instruments, U.K.) at a fixed angle (173°) using a 633 nm laser, which precluded any excitation of the fluorophore. Further analysis of NP size was performed using nanoparticle tracking analysis (NTA) with a NanoSight NS500 (Malvern Instrument, U.K.). For the zeta potential, laser Doppler anemometry was employed using a Zetasizer Nano ZS with DTS1070 folding capillary cells. NP suspensions were sonicated (USC200TH, VWR) for 3 min before measurements. For accurate analysis, a dilution of 1:400 (5/2000 μL) with Milli-Q water was performed to ensure the particle count rate was below 5 × 10^5^ counts per second. Three measurements were performed for each sample, and the data were reported as mean ± SD. Surface morphology analysis of the NPs was investigated using field emission scanning electron microscopy (FE-SEM; JSE-5200, JEOL, Japan). For suspended NPs, 50 μL of nanosuspension in Milli-Q water was added directly onto the surface of a mica slide and left to air-dry prior to preparation for imaging. For atomized pMDI-NPs, samples were directly aerosolized onto either mica or an MCE membrane (1.2 μm pore size; Millipore) within the aerosol deposition apparatus as described above (Section 2.4). Following NP deposition, the mica and MCE membrane samples were placed on an aluminum stub with carbon tape and allowed to dry for 16 h under high vacuum. Prior to FE-SEM imaging, samples were sputter-coated with chromium (Q150 V S Plus, Quorum, U.K.). For direct quantification, predefined masses of NPs were resuspended in Milli-Q water and serially diluted. The fluorescence of the FITC-PLGA NPs was measured using a plate reader (CLARIOstar, BMG Labtech, Germany), and a linear calibration plot was generated by plotting the particle mass against fluorescence.

### 2.6 Dose Deposition on Cell-Free ThinCert Inserts

For NP deposition on a cell insert, the polyester membrane of the cell-free ThinCert insert was covered with a glass coverslip (Ø = 12 mm) and the inset was placed onto the 3D-printed holder in the exposure chamber of the deposition system. Prior to the experiment, pMDI canisters were manually shaken 10 times, sonicated for 90 s, and primed to waste three times. Twenty actuations were performed, and the aerosol deposition factor based on the recovered NP mass from the inset, with and without application of airflow (15 L/min), was calculated. The coverslip surface was rinsed with Milli-Q water (1 mL), and the samples were quantified by fluorescence spectroscopy using a plate reader (CLARIOstar, BMG Labtech, Germany) against a calibration curve for the dispersed particles. A control of a formulation-free inhaler (Milli-Q water, 2% w/w + EtOH, 2% w/w in HFA134a) was also tested. Three repetitions were performed for each experiment with a total exposure time of ∼1.5 min, and the data were reported as the mean ± SD.

### 2.7 Cell Culture Maintenance and RPMI 2650 Multilayer Development

RPMI 2650 nasal epithelial cells were cultured in EMEM supplemented with 10% FBS, 1% l-glutamine, 1% NEAA, and 1% P/S antibiotic mixture, incubated at 37 °C in a humidified 5% CO_2_ atmosphere and subcultured every 5–6 days. For NP studies, ThinCert cell culture inserts (Greiner Bio-One, Austria) were coated with rat tail collagen type I (50 μg/1.13 cm^2^), which was allowed to air-dry prior to seeding RPMI 2650 cells at a density of 3.5 × 10^5^/1.13 cm^2^. Cultures were kept immersed in culture medium for 48 h, after which air–liquid interface (ALI) cultures were developed by removing the apical medium. The cell seeding density and collagen coating concentration had been previously optimized to maximize TEER values and mucus production (Supporting Information Sections S1.4–S1.6). These cultures were maintained for 14 days, replacing the basal medium every 2–3 days to allow cell differentiation in terms of tight junction expression and mucus production as previously described^29,31^ prior to permeability studies.

### 2.8 Cell Layer Integrity Following Aerosol Exposure

To facilitate live cell imaging without the prior need to cut out the insert membrane, potentially damaging the RPMI 2650 epithelial layer, an inverted cell culture method was adopted. The basal side of Snapwell inserts was coated with type I collagen by pipetting 100 μL of collagen solution (50 μg/cm^2^ in 1:1 EtOH/Milli-Q water) onto the membrane surface and allowing it to air-dry for 4 h. The membranes were then gently washed with PBS and 200 μL of RPMI 2650 cell suspension (3.5 × 10^5^ cells/insert) was pipetted on the coated side and allowed to spread evenly over the surface. The cells were allowed to adhere to the membrane by incubation at 37 °C and 5% CO_2_ for 4 h. Excess medium was removed with care using a gauze swab and the Snapwell insert was then placed back into its housing in the usual, upright position with the cells now inverted. Fresh, prewarmed culture medium (400 μL) was added to the apical compartment and 3 mL was added to the basal chamber. The Snapwell was incubated under immersed conditions for 48 h and then transferred to ALI by removing the medium from the basal chamber and maintaining the culture for 14 days to allow cell differentiation, replacing the apical medium every 2 to 3 days.

The NP deposition apparatus was sterilized with 70% isopropyl alcohol, and the procedure was performed under aseptic conditions in a cell culture hood. Before aerosolization, the canisters were shaken, sonicated, and primed, and 10 or 20 actuations per test were carried out. One cell insert at a time was placed onto the holder in the exposure chamber. The RPMI 2650 epithelial layers were used in the aerosol deposition system as either (a) a blank, kept in the chamber for the time of the experiment (30–90 s) without being exposed to the aerosol, (b) a sham, where the cell layer was exposed to an NP-free pMDI aerosol, or (c) a test, where the cell layer was exposed to the PLGA NP aerosol from the pMDI device.

Following the experiment, each insert was snapped into its original housing, placed in a fresh 6-well plate containing prewarmed medium, and prepared for TEER measurement to predict initial cell layer integrity (Section S1.5). For this test, the inserts that achieved TEER > 30 Ω·cm^2^ were further tested with the LIVE/DEAD double staining viability assay to confirm cellular health according to the manufacturer’s instructions. Briefly, the medium was removed from the inserts, and the cells were rinsed with HBSS, after which they were incubated with 100 μL of the assay solution (2 μM calcein AM and 4 μM propidium iodide in PBS) and incubated for 15 min at 37 °C. The cell inserts were then washed free of any excess stain before being immediately transferred into an imaging dish (35 mm with a glass bottom, Ibidi) so that the cells were facing downward and rested on a drop of HBSS to prevent drying prior to imaging by confocal microscopy (LSM 880, ZEISS, Germany).

### 2.9 Transepithelial Transport of FITC-PLGA NPs

For permeability studies, ThinCert 12-well inserts were used. After 14 days of ALI RPMI 2650 cell culture, following seeding on the apical side of the membrane at 3.5 × 10^5^ cells per insert, the cells were exposed to the test formulation as a nanosuspension and via pMDI aerosolization using the deposition system (Section 2.4). For the suspension permeability measurement, an aqueous dispersion of NPs (1% w/v) was diluted 10-fold with HBSS to a final concentration of 0.1% w/v, and the pH of the buffer was adjusted to 7.2 with HEPES (25 mM, final concentration 1% v/v). The cell inserts were rinsed twice with warm HBSS and incubated with HBSS in the apical and basal chambers (15 min, 37 °C, 5% CO_2_) to allow cells to adjust to the transport medium (HBSS + 1% v/v HEPES). The FITC-PLGA NP suspension (250 μL) was added to the apical chamber, and HBSS (1.5 mL) was added to the basal chamber. Samples (200 μL) were collected every 30 min over a 4 h period from the basal chamber and then at 24 h. An equal volume of fresh HBSS was added to the basal chamber at each time point to maintain the sink conditions. The samples were transferred into a black 96-well plate and NP mass in the acceptor chamber was determined by fluorescence spectroscopy (λ_ex_ = 495 nm/λ_em_ = 520 nm) in comparison to a calibration curve for the aqueous dispersion of the particles. The permeation coefficient (*P*_app_ (cm/s)) was calculated using eq 1
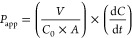
1where *V* is the volume of the receiver chamber (cm^3^), *C*_0_ is the initial concentration of the fluorescent marker (μg/mL), *A* is the surface area of the insert (cm^2^), and d*C*/d*t* is the rate of change of mass of the marker in the receiver chamber (μg/s), in other words, the slope of the regression line obtained by plotting the cumulative mass of the permeated substance collected in the acceptor chamber against time, considering only the linear part of the graph. To confirm that the ThinCert insert membrane was not the rate-limiting step for the permeation process, the permeability of the formulation through cell-free inserts (*P*_B_) was also tested and the epithelial permeability (*P*_E_) was calculated according to eq 2, where *P*_T_ is the total permeability of the whole system.
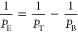
2

Aerosol permeability measurements used a similar setup to the cell aerosol exposure experiments in the deposition system as described for the integrity test (Section 2.8), but with cells being seeded on the apical side of the insert membrane. Following exposure to aerosolized NPs, the ThinCert inserts were transferred into a fresh 12-well plate containing transport medium (1.5 mL) in the basal chamber. Throughout the transport experiments for both NP suspensions and aerosols, cell inserts were incubated at 37 °C in a 5% CO_2_-humified atmosphere. Similar to the suspension permeability test, 500 μL samples from aerosol-exposed cell cultures were collected every 30 min over a 4 h period and at 24 h, with HBSS being replenished at each time point. For the aerosol formulation, the initial dose deposited on each layer (100%) was back-calculated by summing the mass of NPs found in the two ThinCert chambers with that associated with the cells at the end of the transport studies. For both NP suspension and aerosol studies, fluorescence was measured in both compartments at the end of the transport experiment. The donor chamber was washed five times with fresh HBSS (100 μL) to recover the nonpermeated particles and the collected samples were analyzed as described above. The dose associated with the cells, which could decrease the efficient permeability of the particles across the barrier, was calculated following sample collection. Cells were lysed by osmotic shock with 500 μL of Milli-Q water for 1 h followed by multiple freeze/thaw cycles (5 cycles for efficient lysis). The lysed cells were then centrifuged at 18,879*g* for 5 min (Thermo Scientific-Heraeus Pico 17) and the NP content in the supernatant was analyzed.

### 2.10 Nasal Cast and Custom Snapwell Insert Fabrication

**Figure 2:**
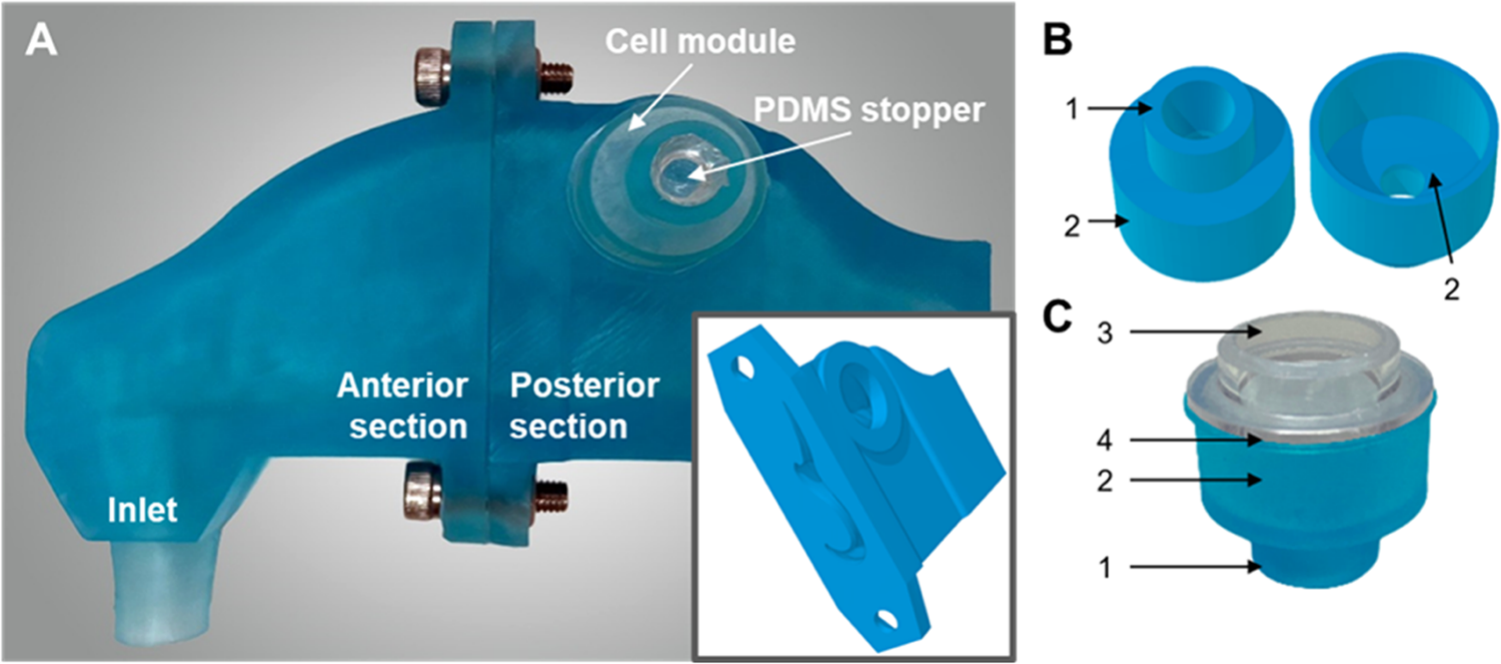
Custom cell module and 3D human nasal cavity model for culture of RPMI 2650 cells and deposition of aerosolized PLGA NPs in the olfactory region. (A) 3D-printed nasal cavity model shows the custom Snapwell cell module fitted into the olfactory region of the cast. The inset shows the 3D model of the posterior section of the cast with the open port to the olfactory region visible. (B) 3D model of the cell module from below and above. (C) A photograph of the 3D-printed module with a Snapwell insert attached: (1) Culture medium port, which is sealed with a PDMS stopper; (2) culture medium reservoir; (3) inverted Snapwell insert for ALI cell culture; (4) interface between Snapwell and printed module, sealed with silicone glue.

To examine the deposition of aerosolized FITC-PLGA nanoparticles onto cells in a replica nasal cavity model, this study utilized the Carleton-Civic standardized human nasal geometry described by Liu et al.^37^ The 3D nasal cast model was split into anterior and posterior segments using SketchUp (Trimble, Inc.), with the posterior section sealed in order to simulate holding breath (i.e., no airflow). A circular port (Ø = 15.6 mm), which was designed to accept a Snapwell cell culture insert, was added in the olfactory region (Figure 2A and inset). Cells were cultured on the basal side of the Snapwell membrane as described below (Section 2.11) and, to maintain ALI cell culture, the inserts were bonded using silicone glue (Silcoset 151) to a custom 3D-printed module, which provided a reservoir of culture medium (Figure 2B,C). Both the nasal cast and cell module were printed on an Anycubic Photon Mono SE printer using clear blue Engineering Like Resin (Eono). Cell modules were sealed with PDMS plugs, which were fabricated by casting a 10:1 mixture of elastomer and curing agent (Sylgard184 silicon elastomer kit) into 3D-printed molds of the culture medium port and curing for 16 h at 60 °C.

### 2.11 Cell Compatibility and Deposition Studies of Aerosolized FITC-PLGA NPs in the 3D Nasal Cast

**Figure 3:**
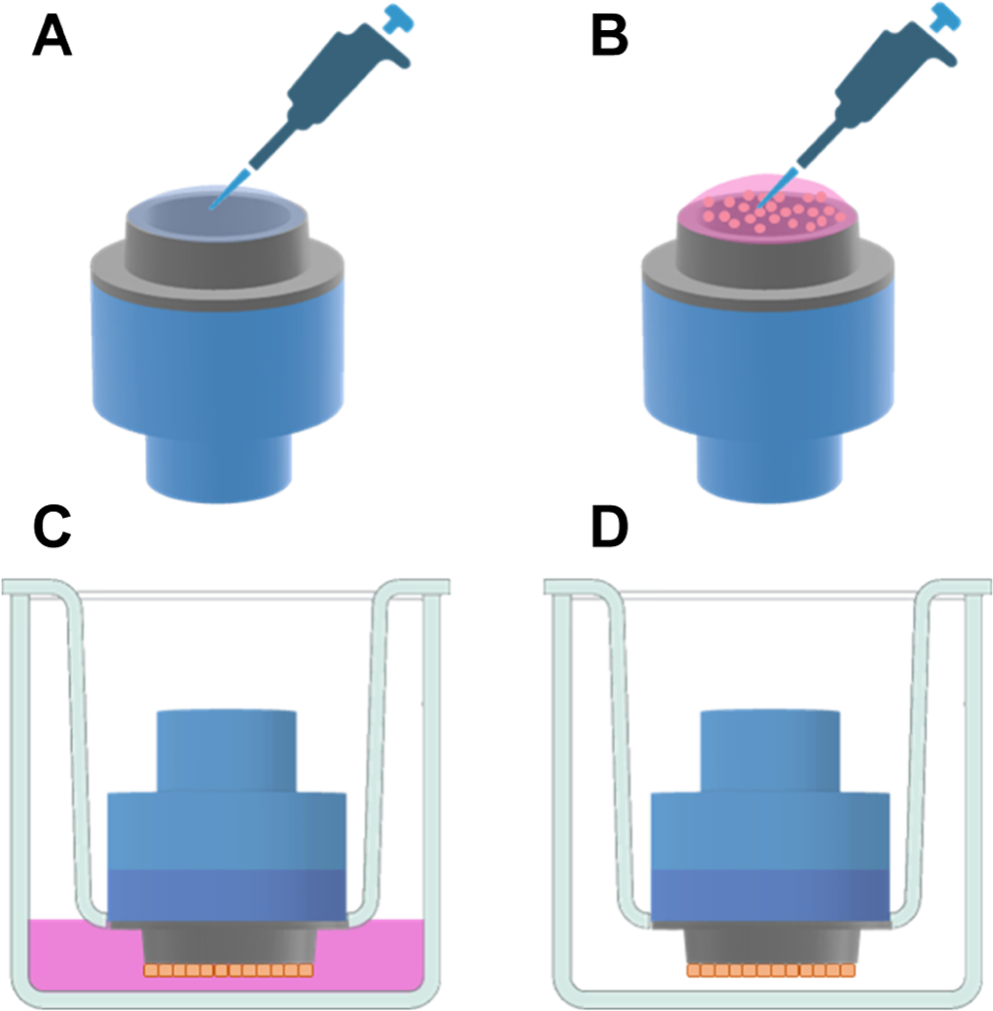
ALI culture of RPMI 2650 cells using the custom cell module. Following collagen coating of the Snapwell membrane (A) and seeding with RPMI 2650 cells for 4 h (B), cells were cultured for 48 h immersed in culture medium (C). The culture medium was removed from the well and retained within the cell module to create an inverted ALI culture (D), which was maintained for 14 days.

The custom Snapwell module (Figure 2B,C) was thoroughly washed with PBS, sterilized under ultraviolet (UV) light in a cell culture hood for 30 min, and then left in the hood for 2 h to air-dry. The module was inverted, and the membrane was coated with type I collagen and seeded with cells as described in Section 2.8 to establish a 14-day ALI culture (Figure 3). Cell integrity and NP formulation deposition experiments were carried out in a stationary nonairflow mode where the custom Snapwell was mounted in its allocated space in the nasal cast (Figure 2A) and 1 mL of fresh transport medium was added into the reservoir. For the cell integrity test, the cell layers were either (a) a blank, kept in the cast for the time of the experiment (90 s) without being exposed to the aerosol, (b) a sham, where the cell layer was exposed to a formulation-free pMDI aerosol within the cast, or (c) a test where the cell layer was exposed to the PLGA NP aerosol (20 actuations) from the pMDI device within the cast. For the deposition studies, 30 actuations were performed to ensure a clear fluorescent signal. Following aerosol exposure, the medium was removed from the assembly reservoir, and the inserts were carefully detached from their support with a fine spatula and transferred into a glass-bottom dish for imaging using confocal microscopy (LSM 880, ZEISS, Germany).

### 2.12 Statistical Analysis

Where applicable, statistical sample variabilities were evaluated using unpaired two-tailed Student’s *t* test or one-way analysis of variance (ANOVA) followed by post hoc Tukey’s HSD analysis using GraphPad Prism 9.4.1 software. All data were expressed as mean ± standard deviation (SD). A value of *P* < 0.05 was considered statistically significant.

## 3 Results

### 3.1 PLGA NP Preparation, Characterization, and Integrity in an HFA134a Dispersion

Fluorescent FITC-PLGA NPs were prepared via nanoprecipitation for formulation in a pMDI. The NP particle size distribution recorded by dynamic light scattering for all prepared batches was within the range of 193 ± 3 nm (*n* = 6), with a narrow PDI of ≤0.1. As fluorescent particles can potentially affect the quality of DLS data, nanoparticle tracking analysis was also employed to assess particle size, with a resultant mean diameter of 167 ± 69 nm (*n* = 5). Particle surface charge was negative within the range of −12.1 ± 2.8 mV (*n* = 6). For the pMDI-NP formulation, initial visual examination revealed a creaming effect due to density differences between the aqueous NP suspension and bulk HFA134a propellant. The water content of the formulation (2% w/w) was the nominal amount required to produce a well-dispersed 0.1% w/w NP suspension, while lower water content (0.5 and 1% w/w) was insufficient, resulting in a significant amount of the formulation being lost due to adhesion to the inner wall of the glass tubes (not shown). EtOH inclusion was also important, and irreversible particle aggregation was observed in its absence in addition to a drastic reduction in particles being released from the device (data not shown).

**Figure 4:**
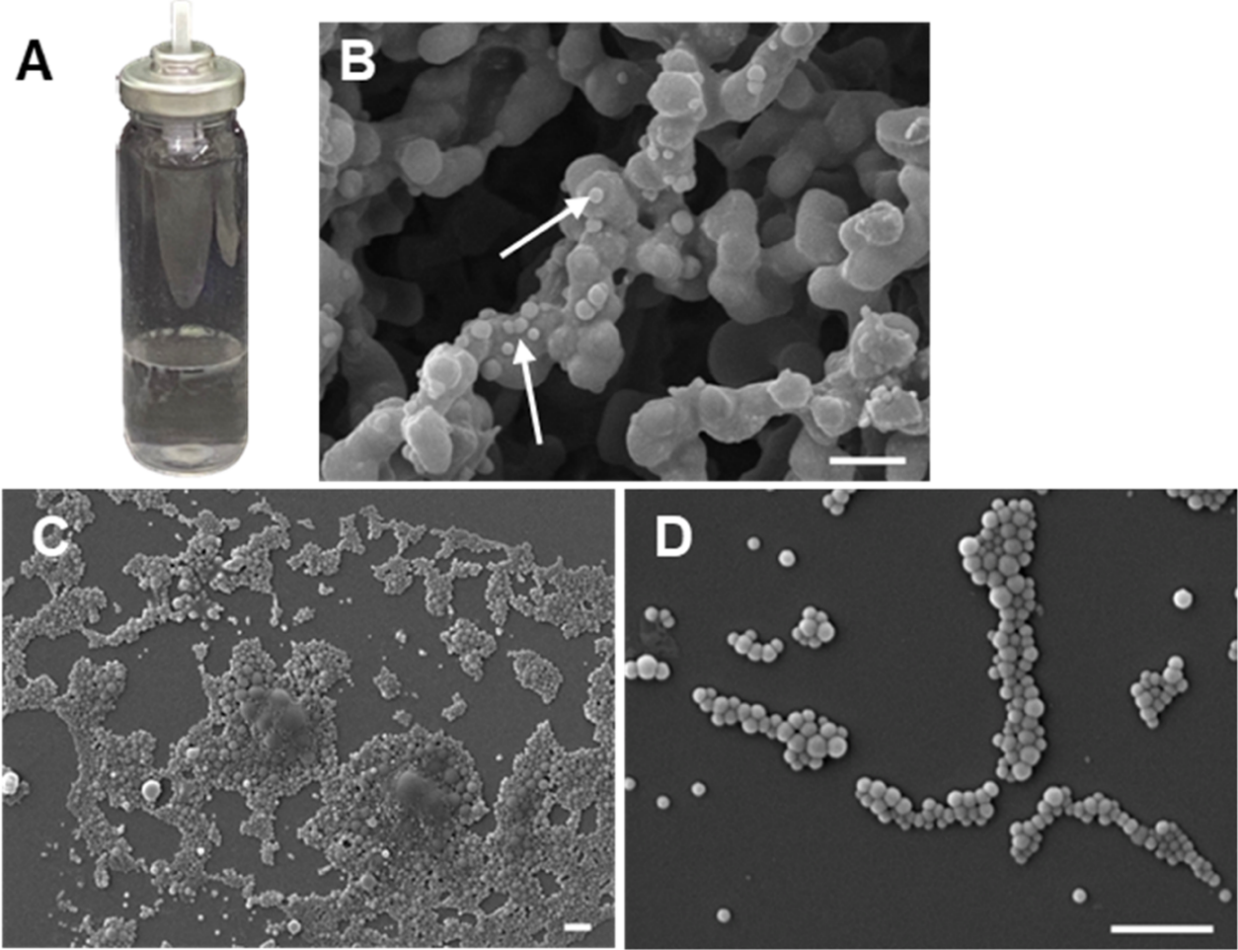
(A) Photograph of the PLGA NP pMDI formulation in a clear poly(ethylene terephthalate) vial for visual examination. (B–D) FE-SEM images of aerosolized NPs from the pMDI device collected on an MCE membrane (B; NPs indicated by arrows) and a mica substrate (C, D) within the aerosol exposure chamber. Scale bars are 1 μm.

The pMDI formulation described in Table 1 was shown to maintain intact NPs following aerosolization while, at the same time, enabling a considerable quantity to be delivered from the pMDI device (Figure 4B–D). Following the pMDI device priming procedure, the canister weight before and after individual and consecutive actuations was measured to ensure the shot-to-shot reproducibility. Using a precise analytical balance, a consistent shot mass of 61.2 ± 0.8 mg was recorded throughout the study, although there was some variation in the emitted dose of FITC-PLGA NPs when different ranges of shot numbers were compared (Supporting Information, Figure S1).

**Table 1:** PMDI Nanosuspension Formulation Composition

	
NP formulation		% w/v	pMDI formulation	% w/w
organic phase	acetone	OP/AP = 1:2	HFA134a propellant	95.9%
aqueous phase^a^	Milli-Q water		NPs	0.1%
polymer	FITC-PLGA	1%	Milli-Q water	2%
surfactant	PVP-K25	0.5%	EtOH	2%
stabilizing agent	PVA	0.5%	total density g/cm^3^	1.207

a A dilution phase of NaCl (25 mM) was used as 5-fold of the aqueous phase for better dispersity and homogeneity of the nanosuspension.

### 3.2 Deposited Dose of pMDI-NP on a Cell-Free Insert within the Aerosol Deposition System

Prior to cell experiments, the delivery performance of the pMDI-NP formulation, in the presence or absence of airflow, was assessed by determining the dose deposited onto a glass coverslip in a cell-free ThinCert following 20 actuations of the pMDI. The main characteristics of the 0.1% w/w nanoformulation are summarized in Table 2. Given the inhaler parameters of a 50 μL metered dose and shot weight of ∼60 mg, the theoretical NP dose in 20 actuations was 1.20 mg, assuming perfect shot-to-shot uniformity (60 μg per shot) of the 0.1% w/w NP formulation. For the deposition apparatus used in this study, the active surface area exposed to the aerosol was ∼10.2 cm^2^ and 11% was covered with the central cell insert (1.13 cm^2^). Hence, if the deposited dose was solely correlated with propellant force, the ideal expected insert dose would be 11% of the total “invested formulation” aerosolized in the 20 actuations. Therefore, the maximum theoretical dose deposited on the cell insert under those conditions would be 0.13 mg, assuming no dose loss following atomization. However, with no airflow applied, 1.8 ± 0.4 μg of the NP dose was collected from the insert surface compared to 1.0 ± 0.4 μg recovery with 15 L/min flow passing through the deposition apparatus. These results are summarized in Table 2.

**Table 2:** Main Features of the Studied PLGA NP-Based Inhaler and the Estimated Delivered Dose to the Cell Insert

pMDI formulation components	Density (g/mL)	%w/w (mg) (μL)	formulation density (g/mL)	1.207
NPs	1.3^a^	0.1 (16.3)	shot volume (μL)	50
H_2_O	1	2 (326) (326)	NPs dose per shot (μg)	60
EtOH	0.791	2 (326) (412)	shot weight (mg)	58–61
HFA134a	1.226	95.9 (15,632) (12,750)	dose number (∼)	270
total mass (mg)		16,300	% of the total NP mass in 20 shots	
NP dose without AF (μg)		1.80 ± 0.37	without AF	0.11%
NP dose with AF (μg)		0.98 ± 0.40	with AF	0.09%

a Density of PLGA; AF, airflow (15 L/min).

### 3.3 RPMI 2650 Cell Layer Integrity Following Aerosol Exposure

**Figure 5:**
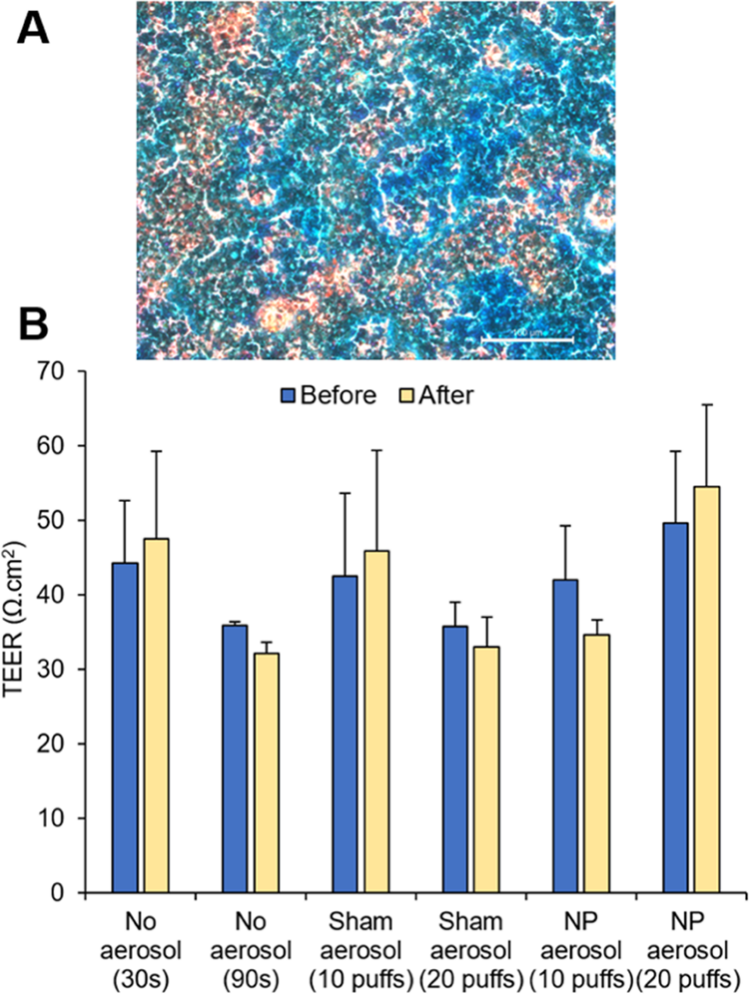
(A) Light microscopic image for RPMI 2650 cells seeded at density (3.5 × 10^5^ cells/cm^2^) upside-down on the Snapwell insert. The mucus secretion was analyzed with Alcian Blue staining. Scale bar = 100 μm. (B) TEER values (mean ± SD, *n* = 2) for RPMI 2650 cell layers inversely seeded on Snapwell inserts before and after the aerosol exposure within the deposition chamber.

To confirm the growth and integrity of the nasal epithelium RPMI 2650 cells when cultured on the underside of Snapwell membranes, cells were assessed for confluence using light microscopy and stained for the secretion of mucus. Figure 5A shows that these inverted culture conditions resulted in a confluent layer of mucus-producing cells, where mucin proteins are stained blue, confirming that the inverted Snapwell culture was suitable for subsequent integrity, deposition, and permeability tests in the nasal cast with the added benefit of being conducive to live cell imaging due to the cells being on the basal side of the membrane.

Prior to further evaluation, it was important to demonstrate that exposure to the aerosol formulation developed in this study was not detrimental to the barrier function and viability of the ALI RPMI 2650 cell layer. Barrier function was evaluated by monitoring the TEER values across the cells; for an epithelium, high TEER values are reliable indicators of the cell layer integrity. However, as RPMI 2650 cells form a rather leaky barrier in comparison to the nasal epithelium in vivo, TEER values >30 Ω·cm^2^ were considered sufficient, as reported in other studies.^22,38^ After aerosol exposure (10 or 20 puffs), cells were gently washed with HBSS buffer, and the TEER was evaluated following the protocol described in Section S1.5. Figure 5B shows that no significant differences were detected under all studied conditions when TEER values pre- and post-aerosol exposure were compared (*P* > 0.05; two-tailed paired *t* test), suggesting that aerosol exposure did not affect RPMI 2650 barrier function.

**Figure 6:**
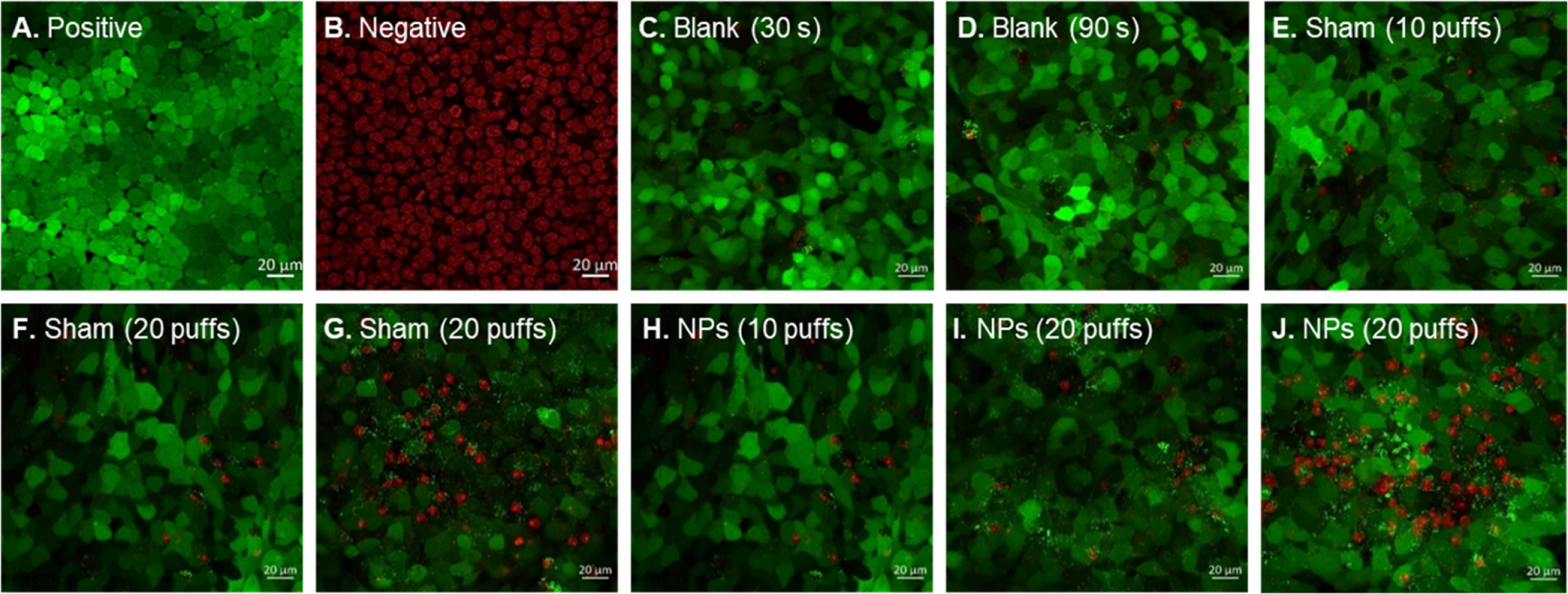
RPMI 2650 cell viability following PLGA NP aerosol exposure delivered via a pMDI inhaler within the aerosol deposition system. Live cells are stained green, while dead cells are stained red. Positive control cells were untreated, whereas negative control cells were cells incubated in 70% EtOH for 30 min prior to staining.

Since the expected low TEER values reported here might not be conclusive on their own for the demonstration of barrier maintenance, the integrity of the cell model was further examined by LIVE/DEAD double staining for cell viability as an alternative measure following aerosol exposure and TEER assessments. Figure 6 compares confocal imaging of the cell layer following different treatments: blank (no aerosol), sham aerosol (formulation-free aerosol), and FITC-PLGA NP aerosol. A confluent layer of fluorescent green cells was visualized when the inverted Snapwell culture was subjected to a 15 L/min airflow for 30 s (Figure 6C) or 90 s (Figure 6D) with no aerosol, indicating an intact and viable cellular barrier under control experimental settings. When 10 puffs of the sham aerosol were applied, a similar undamaged barrier to the blank was observed (Figure 6E), indicating the propellant-driven aerosol had no harmful effect on the nasal cells at 150 mm distance from the insert. While no differences were noticed across sham inserts when applying 10 puffs, the apical damage was higher in some spots than others and between repetitions for the 20 puff-sham (Figure 6F,6G). A similar trend was noticed for the 10 puff-NP aerosols (Figure 6H) and 20 puff-NP aerosols (Figure 6I,J). Collectively, the formulation of FITC-PLGA NPs in an HFA134a propellant-based aerosol was well tolerated by the RPMI 2650 nasal epithelium model. While some evidence of damage to the cell layer was observed, cell viability and barrier integrity, measured by TEER, remained at an acceptable level, suggesting that this formulation could be considered suitable for nasal delivery.

### 3.4 Transepithelial Transport of pMDI-PLGA NPs across the Nasal Barrier

**Figure 7:**
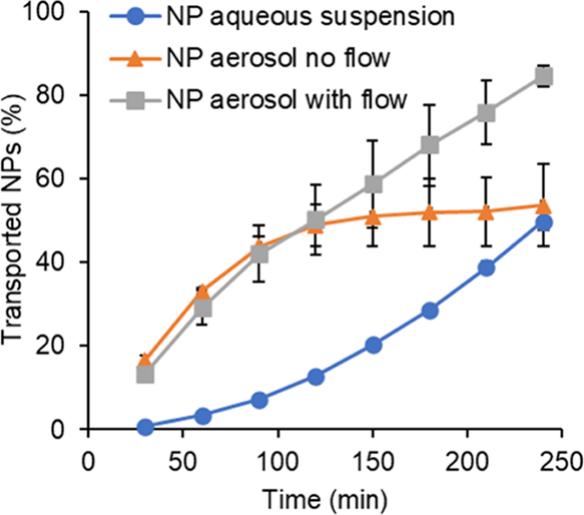
Percentage of PLGA NPs transported across RPMI 2650 cell layers on ThinCert membranes over 4 h following delivery as an aerosol within the aerosol deposition apparatus (Figure 1) with and without a 15 L/min airflow or as an aqueous suspension (mean ± SD, *n* = 3). Data represent the percentage NP mass relative to the total NP mass recovered at 24 h.

**Figure 8:**
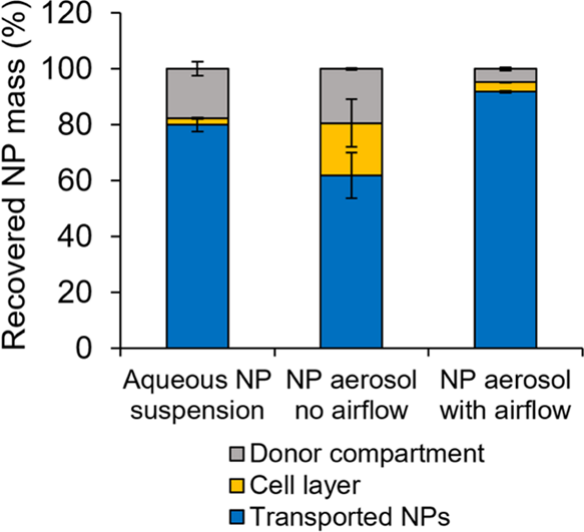
Distribution of PLGA NPs recovered 24 h after exposure of RPMI 2650 cells on ThinCert cell inserts to an aqueous NP suspension or NPs aerosolized *via* a pMDI (mean ± SD, *n* = 3).

Following the successful demonstration of the maintenance of barrier integrity and cell viability following exposure to the aerosolized FITC-PLGA NP formulation, an ALI RPMI 2650 model on ThinCert membranes was used to evaluate the transport profile of the NPs. To achieve this, the transport of NPs in an aqueous colloidal suspension was compared with that of aerosolized NPs, simulating administration of this formulation for intranasal drug delivery. The apparent permeability coefficient, *P*_app_, of the aqueous FITC-PLGA NP suspension was found to be 2.77 ± 0.08 × 10^–6^ cm/s. This parameter, however, was not examined for aerosolized NPs due to the high variability of the localized dose on the surface of the cell insert for the 20 actuated puffs (1.80 ± 0.37 μg without flow and 0.98 ± 0.40 μg with flow, as described in Section 3.2). Although the *P*_app_ values could not be directly compared, it is reasonable to build the comparison between the suspension and aerosolized formulations upon the quantity transported across the cells as a percentage of the total recovered dose from the whole system, as presented in Figure 7. Similar transport profiles were observed for the aerosolized NPs with and without 15 L/min airflow in the first 2 h postexposure, with transport of approximately 50% of the recovered dose achieved. Beyond 2 h, NP transport continued to increase across cells exposed to airflow, whereas transport plateaued for the static condition. For both the NP suspension and aerosolized NPs exposed to airflow, uptake was approximately linear between 2 and 4 h (Figure 7) with the transport rate being similar for the two conditions. As shown in Figure 8, at 24 h postexposure the majority of the recovered dose for aerosolized NPs with airflow was in the receiver chamber (91%) with very low levels recovered from the donor compartment (4.8%) and cell layer (3.4%). However, for aerosolized NPs with no airflow, ∼19% of the total recovered NP dose was retained in the cell layer and an almost equal amount was found in the donor compartment. For the NP suspension, ∼80% transport across the cell layer was achieved after 24 h with the majority of the remaining dose recovered from the donor compartment (Figure 8).

### 3.5 pMDI-PLGA NP Formulation Compatibility and Deposition Studies in RPMI 2650 Cultivated in the Human Nasal Replica

**Figure 9:**
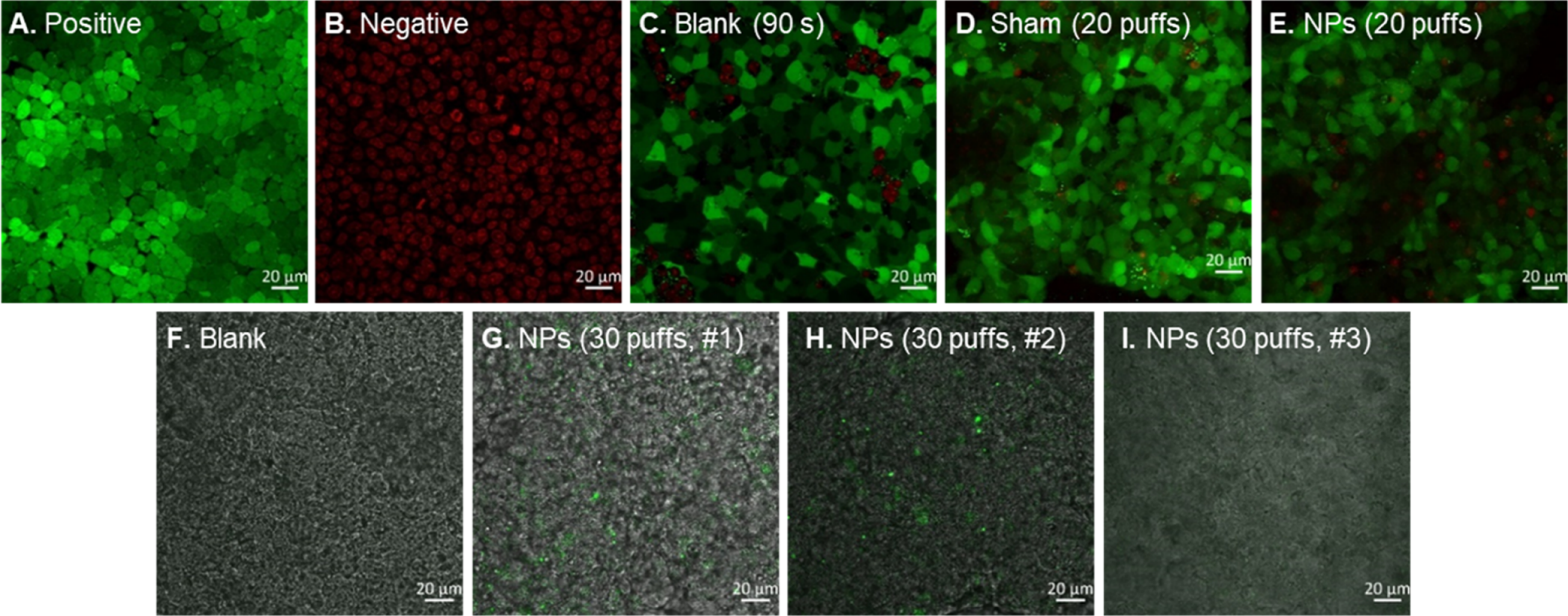
(A–E) Viability of RPMI 2650 cell layers in the olfactory region of a 3D human nasal cavity replica following exposure to aerosolized FITC-PLGA NPs from a pMDI. Viable cells are stained green, while dead cells are stained red. The positive control was untreated cells, while the negative control was cells exposed to 70% EtOH for 30 min. (F–I) Overlay of phase contrast and fluorescent images of ALI RPMI 2650 cell layers in the olfactory region of a 3D human nasal cavity replica showing deposition of three different batches of aerosolized FITC-PLGA NPs.

In this study, a custom Snapwell insert positioned at the olfactory region in a two-section nasal cast was utilized as a proof of concept to demonstrate the deposition of a FITC-PLGA NP pMDI formulation in the targeted region and their minimal adverse effect on the nasal barrier integrity. A normal confluent cell multilayer (Figure 9) was obtained using the custom insert indicating that the resin mold was compatible with the cultured cells and their medium (no color change was observed in the cell culture medium). For the epithelial integrity test, since the TEER measurements were not possible with this system, the LIVE/DEAD double staining protocol was employed instead as previously described in Section 2.8. The confocal micrographs for the 90 s blank (Figure 9C), the 20 puff-sham (Figure 9D), and the 20 puff-sample (Figure 9E) show similar outcomes, with very few dead cells visible in the apical layers. The wall shear stress effect on the nasal cells within the cast was not considered since no respiratory airflow was applied. Following administration of three different batches of aerosolized FITC-PLGA NPs to the cell-containing nasal cast, obvious green fluorescence clearly demonstrated successful particle deposition on the cell layer within the olfactory region of the cast following 30 actuations (Figure 9G–I).

## 4 Discussion

The development of biorelevant testing platforms to examine drug deposition and transport is required to assess the performance of nasal aerosols. Since the efficacy of nasal products is associated with the formulation, delivery device, and administration techniques applied by the patient, in vitro models that allow the simulation of nasal delivery from the generation of an aerosol to the transport of aerosolized particles across the nasal mucosa would ultimately enhance in vitro–in vivo correlations in comparison to conventional in vitro cell models. This study focuses on, first, developing a formulation for nasal inhalation that can achieve effective deposition in the olfactory region and, second, developing an in vitro system to evaluate the performance of this and other intranasal formulations.

Polymeric nanoparticles have been demonstrated to be a suitable drug delivery strategy for the management of CNS disorders via the nose-to-brain pathway due to their small size, large surface area, and tunable physicochemical properties, enabling them to overcome biological barriers and provide targeted/controlled therapy.^39^ In this work, PLGA nanoparticles delivered by a pMDI device were chosen as a potential delivery system for targeting the olfactory region. While NPs are advantageous in producing a highly aerosolized formulation, utilizing a device based on a liquified propellant system such as pMDI could further enhance deep penetration into the nasal cavity toward the olfactory region upon actuation. Therefore, a pMDI was chosen as a device of choice to achieve the targeted deposition of this nanoformulation. To aid subsequent analysis of deposition and transport, fluorescent NPs, labeled with FITC, were prepared by conventional nanoprecipitation. While encapsulation of free fluorescein would have been likely to produce small, ultrabright particles with high dye loading capacity, release/leakage of the dye during in vitro deposition testing may have resulted in misinterpretation of particle distribution. Hence, to avoid this, an alternative “covalent” strategy using FITC-b-PLGA was employed to generate NPs with a diameter of ∼200 nm. Craparo et al. investigated the cellular trafficking in NTBDD for two types of fluorescent PLGA–PEG NPs: Rhodamine B dye-loaded NPs, and Rhodamine B dye-grafted NPs. Like the particles produced in this study, both carriers were of a size suitable for nasal delivery (<200 nm), were monodisperse (PDI < 0.3), and highly biocompatible with olfactory ensheathing cells. The authors concluded that the two labeled systems were useful imaging tools for in vitro/in vivo nose-to-brain studies.^40^ In another study, it was demonstrated that producing ultrafine NPs (<100 nm) was not necessary to achieve efficient delivery across the nasal mucosa and NPs of >100 nm showed good uptake and transferability, suggesting the properties of the NPs generated in this study are suitable for NTBDD.^41^

To aerosolize the NPs from a pMDI device, a formulation of FITC-PLGA NPs in a mixture of water, EtOH, and HFA134a propellant was developed, aiding particle suspension and enhancing the delivered dose. It was crucial, however, to maintain minimum solvent content (water and EtOH) so that particle trajectories and therefore deposition would be driven by their morphological characteristics and not by the formation of solvent droplets containing an NP suspension. Following aerosolization from the pMDI, the particles exhibited excellent integrity and were collected intact in the deposition apparatus (Figure 4B–D). As the NPs were lower in density than the rest of the formulation, a creaming effect was observed (Figure 4A). This effect, however, was expected due to the high mass ratio of the propellent (>96% w/w), which has a density of 1.22 g/cm^3^. Despite the phase separation, the particles were easily redispersed by three to five cycles of manual shaking, and the creaming time was long enough to perform one actuation of the pMDI at a time. Stabilizers such as alcohols are a common strategy to increase the solubility of any surfactants utilized to improve the stability of suspension-based HFA pMDIs.^42,43^ Although no surfactants were added to the propellant in this study, the addition of EtOH as a cosolvent was essential to aid the formation of a physically stable aqueous nanosuspension formulation in the propellant upon shaking the device, hence limiting phase separation prior to actuation. In addition, including EtOH was useful in overcoming the formation nondispersible aggregates of NPs and the poor/uncontrolled emitted NP dose when the aqueous NP dispersion alone was added to the propellant (not shown). However, a minimal EtOH content (2% w/w) was utilized in the system to avoid any potentially damaging effect on the NPs as well as on the nasal barrier.

To assess the influence of airflow on the dose of NPs delivered by the pMDI, aerosolized NPs were collected on cell-free ThinCert inserts in the deposition apparatus (Figure 1) with 0 or 15 L/min airflow. This was performed to determine the likely NP mass that would be deposited on cell layers in subsequent transport studies, with the apparatus proving to be a useful tool to contain the aerosol and measure deposition. In addition, this apparatus was easy to use, sterilize, and disassemble and could be placed in standard cell culture hoods. Compared to the theoretical NPs dose per shot (Table 2), the experiment demonstrated a low recovered dose (0.98–1.8 μg) under both airflow and static conditions, which may be explained by several factors. First, the 90 s exposure time of the inserts to the aerosolized NPs (∼200 nm) may be too short for their effective diffusion or sedimentation. These NPs fall within the accumulation mode of airborne particles, where terminal settling velocities are extremely small according to Anastasio and Martin.^44^ The particles are too large (>50 nm) for atmospheric removal by diffusion, and too small for gravitational settling (1–1000 μm). Hence, this particle category has the longest residence time (days to weeks) if not driven by propellant force. The Stokes–Cunningham law (eq 3) can be used to determine the terminal settling velocity (*V*_t_) of a particle
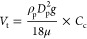
3where ρ_p_ is the particle density (1.2 × 10^6^ g/m^3^), *D*_p_ is the particle diameter (0.2 × 10^–6^ m), *g* is the gravitational acceleration (9.8 m/s^–2^), μ is the dynamic viscosity of air (∼1.846 × 10^–2^ g/m·s at 22 °C, 1 atm), and *C*_c_ is the Cunningham correction factor (∼1.66 at 22 °C). Hence, the NPs in this study would have a terminal settling velocity of ∼2.35 × 10^–6^ m/s, indicating that the experimental time period was insufficient for settling of particles that were not impacted by propellant force. Additionally, the delivered dose of the formulation and dose-to-dose homogeneity may have been affected by the two-phase formulation in the inhaler; differences in the aerosolized dose density and interfacial tension between the formulation and the propellant phases, which impact the wetting properties of the dispersed droplets and the ability to form a homogeneous emulsion, could cause the theoretical and experimental “invested dose” to differ. The water solubility of HFA134a is low (2220 ppm at 25 °C)^45^ and we report a two-phase formulation of the propellant and the aqueous nanosuspension. The tension of the propellant-water interface, which plays a vital role in stabilizing the suspended formulation, was not measured in this study. However, Peguin et al. have determined its value to be 33.5 ± 0.3 mN/m experimentally and 30.8 ± 10.7 mN/m by molecular dynamic computer simulation.^46^ At such a low value, the dispersed water droplets produced within the propellant when shaking the device are more likely to rapidly coalesce, prompting phase separation. Hence, with the canister in the upright position, the less dense nanosupension may be distant from the metering chamber when the device is actuated, affecting the uniformity of NP delivery. As a result, it is possible that some actuations of the device resulted in puffs that were pure propellant.

Unlike nebulizers, pMDI devices generate very low to zero liquid output, especially when the content of nonvolatile solvents with respect to HFA propellant is relatively low (e.g., 2% w/w EtOH in this study). Therefore, the aerosol-to-cell delivery in the exposure chamber would be predominantly driven by, first, the force of the propellant and, second, by the single-particle characteristics and motion i.e., particle impaction and sedimentation mechanisms rather than by a cloud of dense droplet motions. The latter mechanism was studied by Lenz et al. using a vibrating mesh nebulizer for drug delivery to an ALI culture of A549 human pulmonary epithelial cells.^47^ Within that system, three delivery phases for nebulized fluorescein solutions were described. First, a narrow and dense droplet cloud was generated, which decelerated at the bottom of the chamber. Second, the cloud was uniformly distributed around the chamber with the most concentrated mist occurring near the bottom. Finally, the mist deposited onto the cell inserts at the bottom of the chamber, with 84% of the theoretically expected amount depositing on the A549 cells based on the fractional surface area occupied by the inserts.^47^ It would be extremely unlikely for the pMDI formulation described in this study to achieve a similar dose deposited onto the insert or to follow the delivery phases suggested by Lenz et al., where nebulized formulations with relatively large droplet sizes (mass median aerodynamic diameter 4–6 μm) with high sedimentation rates were tested.^47^ However, one would expect that the application of airflow would greatly improve the dosing onto the cell insert. In fact, despite the relatively high variabilities between the runs, a consistent trend was observed with a relatively lower dose achieved using flow compared to static conditions. This could be due to the dose deposition being predominantly propellant-driven and the main particle deposition mechanisms, sedimentation, and diffusion occurring more effectively in a low airflow environment, which is physiologically preferable and relevant to the deep posterior regions in the human nasal cavity where the targeted olfactory region is located.

Despite the relative scarcity of in vitro deposition systems for testing the deposition and transport of nasally delivered formulations, comparable studies have investigated the influence of airflow, NP concentration, spray volume, and positioning of cell inserts on the deposition of pulmonary formulations. Using a HandiHaler device, Hein et al. showed a 2.5-fold increase in the dose of 2–5 μm budesonide particles collected on Snapwell inserts when a short ventilation flow of 6 L/min (2 s) was applied in comparison to 60 L/min (3 s).^48^ Using a deposition system composed of a desiccator and eight ThinCert inserts seeded with Calu-3 cells or coated with porcine tracheal mucus, Cingolani et al. examined the deposition of dry powders aerosolized by a PennCentury Insufflator. The center insert, positioned directly under the device, received a much higher dose (∼3.2 μg salbutamol and ∼11.0 μg indomethacin) compared to six peripheral inserts (mean of 0.85 μg salbutamol and 0.93 μg indomethacin per insert). The authors suggested that inertial impaction was the predominant deposition mechanism for particles with diameters >100 μm onto the central insert, while sedimentation of smaller particles following airflow occurred for the peripheral inserts.^49^ The geometric pattern of the inserts in such systems has also been shown to affect the deposited dose. For liquid aerosols generated *via* a MicroSprayer, a higher deposited dose was obtained with a higher spray volume, and a dose variability up to 72% between the inserts was reported when they were arranged close to the center of the deposition support,^50^ similar to the variability found in this study between three repetitions. In a study using nebulized TiO_2_ NPs, it was shown that by halving the NP concentration and doubling the nebulized volume, a similar NP mass was deposited on cultured rat alveolar macrophages. However, significantly less variation in the delivered dose was observed between the cell inserts in the system, and no increase in the NP exposure time was required (5 min for both formulations).^51^ These studies demonstrate that, in general, in vitro cell aerosol exposure systems lack control over the deposited mass of formulation and result in a low deposition of drug, which can require days to weeks of exposure to achieve a considerable biological response.^52^ Similar to previous reports, it can be concluded that a number of different factors could influence the delivered dose of NPs to the cell inserts in the aerosol deposition system developed in this study. Nonetheless, the presented conditions were pursued for further testing since a detectable dose of FITC-PLGA NPs was recovered from the inserts within a short exposure time.

To assess the compatibility of the FITC-PLGA NP aerosol with nasal epithelial cells, the deposition apparatus (Figure 1) was used to accommodate a single insert with a 14-day ALI culture of RPMI 2650 cells. This facilitated the in vitro testing of epithelial tolerance to the PLGA NP pMDI combination and subsequent particle uptake and transepithelial transport across the RPMI 2650 cell barrier. TEER measurements are broadly used as an index of the integrity of in vitro epithelial cultures. Various TEER values have been described in the literature for ALI RPMI 2650 with reported acceptable thresholds ranging between ∼130,^53^ ∼66,^54^ ∼ 40,^28^ and >25 Ω·cm^2^.^22^ Throughout this study, the RPMI 2650 epithelial model exhibited TEER values >30 Ω·cm^2^ (Figures S2 and 5B), which is low in comparison to the in vivo nasal epithelium, but similar to values reported for these cells in the literature as outlined above. This is, however, not surprising since the evaluation of RPMI 2650 monolayer integrity by reliance on TEER values has been reported to be difficult due to the “leaky” characteristics of this multilayer nasal epithelium model.^22^ However, in future studies it may be possible to increase the patency of the RPMI 2650 barrier by media supplementation, for example, activating Wnt signaling as demonstrated in a cell culture BBB model.^55^

To further determine the suitability of the ALI RPMI 2650 culture for in vitro studies, additional parameters to TEER were also examined, including tight junction marker expression (ZO-1 and E-cadherin; Figure S5), macromolecular permeation (Figure S7), and mucus production (Figures S3 and 5A) confirming the establishment of a biomimetic epithelial barrier. Staining for tight junction markers ZO-1 and E-cadherin (Figure S5) was comparable to that demonstrated in previous studies,^27−29,56,57^ indicating successful barrier formation. The ALI RPMI 2650 cells also stained positively for mucus secretion, although rather than a homogeneous covering, mucus production varied across the cell layers (Figures S3 and 5A). Differences in mucus secretion may be a result of variations in the thickness of the barrier, which consisted of multilayers of cells, or potentially the existence of subclones within the population with differing expression levels of mucins. Such variations in RPMI 2650 mucin staining have been shown in other studies with this cell line.^33,58^ Finally, the permeation of two fluorescent markers, fluorescein sodium and FITC-dextran, across ALI RPMI 2650 cultures was studied. Apparent permeation coefficients of these two molecules were in agreement with literature values (Section S2.6 and Figure S7). Combined, these data confirmed the establishment of a biomimetic epithelial barrier.

To confirm the compatibility of the FITC-PLGA NPs with this epithelial model, the metabolic activity of RPMI 2650 cells was assessed after 4 or 48 h of exposure to NP suspensions. No detrimental effect on cell activity was observed (Figure S6) demonstrating the cytocompatibility of this formulation, in agreement with previous studies which examined the effects of PLGA NPs on RPMI 2650 cells.^59,60^ Suitability for intranasal delivery was demonstrated by confirming FITC-PLGA NP uptake by the cells using confocal microscopy (Figure S8) and flow cytometry (Figure S9). To assess the biocompatibility of FITC-PLGA NPs aerosolized by a pMDI device, 14-day ALI cultures of RPMI 2650 cells were exposed to NP aerosols in the deposition apparatus, and their TEER and viability were then examined. No significant changes in TEER values were observed when cells were exposed to either 10 or 20 actuations from a sham or NP-containing pMDI (Figure 5B), indicating that the barrier function was unaffected by aerosol exposure. On examining cell viability following aerosol exposure, a comparable ratio of viable and dead cells was observed in both sham aerosol and NP aerosol groups when 10 or 20 actuations were performed (Figure 6E–J), with fewer dead cells in control cultures which were placed in the deposition apparatus with airflow but no aerosol exposure. It is, perhaps, unsurprising that a propellant-driven aerosol falling perpendicularly onto the cell layer at a relatively close distance and high particle velocity might have some deleterious effect on the biological barrier. However, for the aerosol formulation in this study, the effect was limited to a few dead cells in the uppermost cell layer and only when subjected to a high dose number (20 actuations) over a long exposure time of 90 s (Figure 6J). The formulation of noninertial nanoscale particles with a composition of more than 96% w/w of rapidly evaporating HFA134a propellant minimizes possible damage caused by forceful aerosol impaction onto the cells. Similar to this study, albeit under different experimental settings, other research groups have described a maintenance of the integrity of respiratory cell models after exposure to inhalable formulations. Using a VITROCELL cloud system, Leroux et al. nebulized a surfactant (extracted from pig lungs) over rat alveolar macrophage cells prior to their exposure to TiO_2_ NPs. The authors stated that the surfactant modulated the physicochemical properties of the NPs preventing any potential cell toxicity.^51^ Pozzoli et al. confirmed the unaltered permeation properties of an RPMI 2650 cell layer seeded on Snapwell inserts following treatment with six sprays of HBSS solution using a VP3 Aptar nasal pump to simulate the nasal deposition process of budesonide nasal suspension (Rhinocort, AstraZeneca).^58^ No difference in the translocation of the marker fluorescein sodium across the barrier was found compared with the untreated cells. Cingolani et al. reported a drop in TEER of a Calu-3 cell layer when treated with one 0.5 mL puff of ambient air or with salbutamol sulfate dry powder delivered via a PennCentury insufflator at a distance of 200 mm from the cell inserts. However, the barrier permeability properties were maintained after exposure and the transepithelial flux of the Lucifer yellow marker was within the acceptable range for the Calu-3 cell model.^49^ Similarly, the data in this study, obtained using two evaluation methods, suggest that neither the aerosolized formulation nor the Snapwell insert handling during the deposition process had a significant adverse effect on the RPMI 2650 cells and the nasal barrier maintained its integrity after exposure to the HFA134a-based pMDI aerosol. While 20 actuations on occasion resulted in a slight increase in barrier damage, as evidenced by LIVE/DEAD staining, this was limited to a few apical cells within the multilayer. As such, further biological testing utilized 20 puffs of the pMDI to ensure that a measurable mass of NPs was deposited on the RPMI 2650 surface.

The transport of FITC-PLGA nanoparticles across the RPMI 2650 nasal barrier was investigated by using an aqueous suspension in comparison to the aerosolized pMDI formulation. The normalized NP transport rates exhibited relatively high values for the three tested delivery conditions, up to 50% of the recovered dose for suspended particles and aerosolized NPs with no airflow, and up to 80% of the recovered dose for aerosolized NPs with airflow (Figure 7). It is a common notion that nanoparticles enhance the transport of encapsulated cargoes across biological barriers compared to free drug, where the rate of uptake varies depending on the drug properties as well as cell membrane characteristics.^61^ Such transport enhancement was confirmed in this study considering the nanoparticles in all three delivery conditions were detected in the receiver compartment at 30 min following dosing. Similarly, Albarki et al. reported ∼2.5% transport across both olfactory and respiratory nasal mucosae within the first 30 min of incubation with a dispersion of 60 nm PLGA NPs. The uptake was reduced to 1.8 and 1.4% for olfactory and respiratory tissues when 125 nm PLGA NPs were instilled, with NP translocation being dependent on tissue thickness.^41^ Utilizing ALI RPMI 2650 cell line and a nebulized formulation of budesonide solution, Pozzoli et al. demonstrated ∼48% permeation across the barrier at 60 min postatomization incubation.^58^ As mentioned previously, these data suggest the importance of the physical form of the formulation, i.e., aerosolization instead of instillation, when investigating inhaled formulations to obtain realistic cellular transport rates.

Beyond the 30 min time point, differences in transport profiles were observed for aerosolized NPs under static conditions in comparison to the aqueous NP suspension and aerosolized NPs with 15 L/min airflow. While the latter two formulations approximated to linear NP transport, the uptake of aerosolized NPs began to plateau after ∼2 h when delivered with no airflow. This might indicate that sink conditions were not maintained in this situation due to NP accumulation in the receiver chamber, hindering efficient transport after this time point compared to the slower moving hence less accumulated suspended particles, indicating that equilibrium may have been reached across the ALI cell layer. Another reason could be because of the depletion of NP mass on the cell surface or NPs being trapped within the mucus layer, decreasing the apical-basal gradient, and hence, no further accumulated doses were attained after this point. Given the expected variabilities in delivered NP dose when aerosolized, head-to-head transport comparison with and without airflow might not be conclusive. However, the applied airflow may have physically forced the NPs into the mucus layer on the ALI culture, improving diffusion through the epithelial barrier, whereas they may have been more loosely associated with the cells in the absence of airflow. In addition, complex and unpredictable interactions may have occurred between the NPs and the epithelium due to the supramolecular arrangement of the PVA/PVP-25 surfactants on the particle surfaces, which might have affected the interaction of the NPs with the mucus barrier. Although the delivered formulation was identical for both conditions, different conformations on the molecular level are possible. NP association with the nasal mucosal barrier was also described for different muco-adhesive and muco-penetrating nanosystems by Clementino et al. The authors emphasized first the presence of multiple permeation-enhancing constituents for efficient nasal epithelium transport. Second, they demonstrated that enhanced nasal absorption could be prompted by NP degradation via nasal enzymes, thereby inducing drug release into the nasal tissues.^62^ While the latter scenario does not represent the system in this study, the former is a valid explanation. In fact, PVAs (75–95% hydrolyzed) have been reported as muco-penetrating polymers that could aid NP motility in the mucus when noncovalently attached to particle surfaces.^63^ Thus, specific surface modifications, as well as NP diameter in the formulation reported in this study, may be expected to achieve further enhancement in NP transport across the nasal mucosa.

An enhanced permeation performance of aerosolized NPs was observed when a 15 L/min airflow was applied (Figure 7). The respiratory airflow, in both static and oscillatory patterns, exposes the nasal epithelium to wall shear stresses which have been shown to cause significant yet temporary structural and functional alterations of the ALI-human nasal epithelium.^64,65^ It was reported that the main response to such stresses is an increase in epithelial cell mucus secretion (mucins and water) and, hence, a decrease in the transport rate across the mucosa and a simultaneous increase in NP accumulation on/within the cell layer may be expected. This impact, however, was subject to the duration of the stress stimulus, which was >15 min, rather than the magnitude of the wall shear stress.^65^ Since the airflow exposure time required to perform 20 actuations in this study was about 90 s, it is unlikely that further mucus secretion by the cell layer was stimulated. Instead, it is more likely that a drying effect of the airflow reduced the first physical barrier, the mucus, enhancing the diffusivity of the aerosolized particles in the flow state as demonstrated in Figures 7 and 8. It is also possible that exposure of ALI RPMI 2650 cells to aerosolized NPs under airflow compromised the integrity of the barrier. If this was the case, then the likelihood is that the direct airflow within the deposition apparatus, which did not mimic the airflow patterns caused by the complex internal anatomy of the human nasal cavity, was the causative factor. Data shown in Figures 5 and 6 indicate that exposure of the cells to 10 to 20 actuations of aerosolized NPs did not grossly affect barrier function or cell viability, indicating that formulation itself is unlikely to have been directly responsible for any barrier degradation. In comparison to the suspended NPs, the aerosol formulation enhanced the transport rate through the nasal cells at all time points over the 4 h study. Indeed, the type of applied test formulation has been reported to have a great influence on the permeability outcomes in both nasal and pulmonary cell models. For instance, the dry powder form of ketoprofen-loaded microparticles achieved better permeability across an ALI RPMI 2650 culture than solution and dispersion forms.^66^ In another study, a large transport increase (>120-fold) of two different dyes was attained across the tighter Calu-3 lung epithelial cell barrier when using the DP-4 Dry Powder Insufflator and MicroSprayer IA-1C Aerosolizer as delivery devices compared to exposure of the cells to the dyes in bulk solution.^67^

In this study, the superiority of the aerosolized NPs in comparison to an aqueous suspension could be explained by the direct deposition of FITC-PLGA NPs onto the cell layers, prompting concentration-dependent transepithelial transport. For suspensions, the required sedimentation time for the NPs to come into direct contact with the epithelial cells further extends their diffusivity path, hence 18% NP mass remained in the donor compartment fluid 24 h after exposure (Figure 8). These findings emphasize the fact that pipetting the formulation to fully cover the cell culture rather than aerosolization for permeation testing would ultimately provide some bias, as it does not consider the clinical conditions of the aerosolization process and the nasal epithelium interface with the atmospheric air. Uneven distribution, with some cellular areas having higher particle concentrations than others, may occur for suspended formulations,^22^ especially when some particle aggregates are present. This may explain the deviation from the linearity of the regression line for the accumulated mass of the permeated NPs against time in the case of the aerosol in this study. Nonetheless, the exposure of an ALI RPMI 2650 culture to aerosolized FITC-PLGA NPs within the deposition apparatus described in this study, and their subsequent uptake, demonstrate a formulation and testing approach that holds promise for intranasal drug delivery. Delivering a dry nanoparticle powder to the nasal epithelium may overcome drawbacks of liquid formulations such as flooding of the turbinates and subsequent clearance of drug. Additionally, the nanoscale dimensions of the formulation and propulsion into the nasal cavity also have potential in delivering the formulation to hard-to-reach areas such as the olfactory region, with consequent implications for NTBDD.

To investigate the applicability of this FITC-PLGA NP pMDI formulation for olfactory targeting and delivery, an ALI RPMI 2650 epithelial culture was placed in the olfactory region of a 3D human nasal cavity model, and the NP formulation was delivered under static conditions via the nostril inlet (Figure 3). These conditions mimic NP delivery while the patient holds their breath or breathes through their mouth rather than 15 L/min airflow, which mimics shallow nasal breathing. This proof-of-concept study demonstrated that the viability of the RPMI 2650 cells was largely unaffected by exposure to 20 actuations of the pMDI sham or NP formulation (Figure 9D,E), with fewer dead cells than the corresponding conditions within the aerosol deposition apparatus (Figure 6F,G,I,J). This is likely due to the reduced velocity of the particles when contacting the cell layer following diffusion and impaction under static conditions within the olfactory region as opposed to direct impaction under airflow at 15 L/min within the glass deposition apparatus. Thus, this more biomimetic in vitro deposition model, which mimics the in vivo delivery of this NP formulation, suggests that aerosolized PLGA NPs are likely to be cytocompatible with the olfactory epithelium. Furthermore, deposition testing of three different canisters of the FITC-PLGA NP pDMI formulation within the nasal cast demonstrated delivery of the fluorescent NPs to the ALI RPMI 2650 cells within the olfactory region (Figure 9G–I). Hence, the NPs were able to penetrate beyond the narrow nasal valve and achieve successful olfactory delivery further demonstrating the potential of an NP-*co*-pMDI nasal delivery system for NTBDD applications. The bright green signal also suggests that a considerable number of NPs were delivered, although there are differences between F1 and F2 (Figure 9G,H) and F3 (Figure 9I) that may result from variation in the emitted dose from the three different pDMI inhalers. It is established in the literature that, for suspension pMDI formulations, shot-to-shot variability can be expected due to the presence of varying numbers of particles in each atomized droplet.^68,69^ This phenomenon may explain the differing apparent deposition from these three FITC-PLGA NP formulations, and some variation was indeed observed when the emitted dose from the pMDIs was investigated (Figure S1). Nonetheless, like the aerosol deposition apparatus, the cell-containing nasal cast introduces the advantages described above for aerosolized NP delivery but with additional nasal morphological features. Hence this offers a model for in vitro nasal drug delivery research which combines in vivo characteristics with the benefits of in vitro testing. While a variety of in vitro models representing the upper airways have been described in the literature using a typical human nasal replica^70,71^ or cell-based systems,^32,33,58^ the model developed in this study is the first, as far as we are aware, to combine nasal mucosal cells within a detailed morphological replica of the human nasal cavity concomitantly.

## 5 Conclusions

In this study, fluorescent PLGA NPs (∼200 nm) were prepared and incorporated into an HFA134a-based pMDI device to target the olfactory region in a human nasal cavity for potential NTBDD applications. The developed formulation was easily redispersed after manual shaking, and the NPs were found to be respirable and intact following aerosolization. RPMI 2650 human nasal epithelial cells were chosen as an epithelial model for in vitro testing of the pMDI-NP formulation. Under air–liquid interface growth conditions, a thick, multilayered, mucus-producing barrier was formed with expression of ZO-1 and E-cadherin tight junction proteins and TEER values which were similar to similar studies in the literature. An aerosol deposition apparatus was established and shown to be a valid system for in vitro testing of aerosolized medicines, enabling the whole process of aerosol drug delivery to be assessed, including aerosol generation, deposition onto RPMI 2650 cells, permeation across the cell layers and examination of the integrity of the epithelial model following aerosol exposure. In this system, the pMDI-aerosolized PLGA NP formulation was shown to be cytocompatible and did not negatively impact the barrier properties of ALI RPMI 2650 cultures. High permeation rates of the PLGA NPs were observed across the nasal cell layers for the three tested delivery conditions (aqueous NP suspension and NP aerosol with and without airflow), which may have been a result of the relatively leaky nature of the RPMI 2650 barrier. However, the NP transport study demonstrated the superiority of the aerosol NP formulation over the NP suspension, with the highest transport rate obtained for aerosolized PLGA NPs with the application of 15 L/min airflow.

By incorporation of an ALI culture of RPMI 2650 cells within a 3D human nasal cast model, it was demonstrated that aerosolized PLGA NPs can be delivered to the olfactory region of the nasal cavity while maintaining epithelial cell viability, thus providing an in vitro model for aerosol delivery to nasal cells under physiologically relevant conditions. Such a system ultimately paves the way for more accurate in vitro screening of nasally inhaled formulations in terms of simultaneous nasal regional deposition, cellular uptake, and transport, the most realistic end point for aerosol delivery, in a more predictive manner than either liquid–liquid interface cell cultures or other ALI models which lack the element of nasal morphology. Hence, this study provides preliminary evidence of the suitability of aerosolized polymer nanoparticles for intranasal drug delivery, including NTBDD, and presents an improved in vitro model for the screening of NTBDD formulations.

## Data Availability

All relevant data are available in the published article and its Supporting Information.

## Supporting Information Available

Methods for the generation of FITC-PLGA nanoparticles, determining the emitted dose of nanoparticles from a pMDI device, development and characterization of an RPMI 2650 nasal epithelial model, and their uptake up of FITC-PLGA nanoparticles; results showing the emitted dose of nanoparticles, characterization of RPMI 2650 cultures (TEER, mucus secretion, tight junction staining and permeability), compatibility of FITC-PLGA nanoparticles with RPMI 2650 cells, and uptake of nanoparticles by RPMI 2650 cells (PDF)
