# Supplementary material for: A Cell-Based Nasal Model for Screening the Deposition, Biocompatibility, and Transport of Aerosolized PLGA Nanoparticles
Source: Mol Pharm. 2024 Feb 9;21(3):1108–24. doi: 10.1021/acs.molpharmaceut.3c00639 (PMC10915796; doi:10.1021/acs.molpharmaceut.3c00639)
Supplement: Supplementary file 1 — mp3c00639_si_001.pdf [file mp3c00639_si_001.pdf]

Supporting information for:

A Cell-Based Nasal Model for Screening the Deposition, Biocompatibility and Transport of Aerosolized PLGA Nanoparticles

Aida Maaz[1], Ian S. Blagbrough[1] and Paul A. De Bank[1,2,3]*

[1]Department of Life Sciences, [2]Centre for Therapeutic Innovation, and [3]Centre for Bioengineering & Biomedical Technologies, University of Bath, Bath BA2 7AY, U.K.

*Corresponding author: p.debank@bath.ac.uk; +44(0)1225384017.

S1. Materials and Methods

S1.1. Materials

For FITC-PLGA NP synthesis, poly(lactide-co-glycolide)-fluorescein (FITC-PLGA; lactide:glycolide 50:50, MW 10-20 kDa), poly(1-vinyl-2-pyrrolidone) Kollidon® 25 (PVP-K25), and sodium chloride (NaCl) were purchased from Sigma-Aldrich (Germany), and polyvinyl alcohol (PVA; 88% hydrolyzed, MW 22 kDa) was purchased from Acros Organics (Belgium). For cell culture and cell experiments, RPMI 2650 cells were purchased from ECACC (Cat. No. 88031602; UK), Eagle's minimum essential medium (EMEM) with L-glutamine, heat-inactivated foetal bovine serum (FBS), non-essential amino acids solution (NEAA), penicillin/streptomycin (P/S) antibiotic solution, rat tail collagen type I solution, fluorescein sodium salt (Flu-Na), fluorescein isothiocyanate–dextran (FITC-Dextran, MW 4 kDa), Alcian Blue 8GX, Entellan™ new, and Hank's balanced salt solution (HBSS) were purchased from Sigma-Aldrich (Germany). SlowFade™ Diamond Antifade Mountant with DAPI, phosphate buffered saline (PBS), and 4-(2-hydroxyethyl)-1-piperazine-ethanesulfonic acid (HEPES) were purchased from Thermo Fisher

Scientific (UK). ThinCert® (PET) 12 well culture inserts (0.4 µm pore size, 1.13 cm^2 surface area) were purchased from Greiner Bio-One (Austria).

S1.2. FITC-PLGA NP preparation.

Fluorescent PLGA NPs were produced at 20 °C by nanoprecipitation using miscible organic and aqueous solvents. A 1% w/v solution of FITC-PLGA in acetone (1 mL) was added to 2 mL of an aqueous solution of PVA and PVP-K25 (both 0.5% w/v) at 0.25 mL/min under continuous magnetic stirring (400 rpm). The colloidal suspension was rapidly diluted 5-fold with NaCl solution (25 mM) and continuously stirred for 2h to ensure evaporation of the acetone. FITC-PLGA nanoparticles were collected and separated from residual surfactants by three cycles of centrifugation (Beckman J2) at 20,000 rpm using fixed angle rotor JA-20.1 for 30 min at 20 °C. After each cycle, the pellet was washed with Milli-Q water. The final pellet was resuspended in 3 mL of Milli-Q water, filtered through cellulose filter paper to remove any accidental aggregates, and then lyophilized by first snap freezing with liquid nitrogen and then freeze drying for 16 h (Lyoquest-55, Azbil Telstar Technologies, Spain).

S1.3 Emitted dose of FITC-PLGA NPs

A pMDI canister was shaken 5-10 times, sonicated for 90 seconds, and primed to waste three times. The NP output from the canister was then determined from six groups of five shots by firing the formulation into a sealed glass vial, which was left untouched for 15 min following firing. The vial was then rinsed thoroughly with MeCN to dissolve and recover the released particles. The collected solutions were concentrated to 1 mL and the fluorescent intensity, indicating the delivered NP dose, was quantified using a plate reader (CLARIOstar, BMG Labtech, Germany) against standard solutions of known masses of FITC-PLGA NPs dissolved in MeCN. The dose recovered from the nozzle of the actuator was also determined. The device was oriented vertically

during spraying to mimic patient inhalation and samples were taken from the beginning of the canister life (between 4 and 33 shot numbers).

S1.4. Cell culture maintenance and RPMI 2650 multilayer development.

RPMI 2650 nasal epithelial cells were cultured in EMEM supplemented with 10% FBS, 1% L-glutamine, 1% NEAA and 1% penicillin/streptomycin antibiotic mixture, and incubated at 37 °C, in a humidified 5% CO_2 atmosphere. Cells were sub-cultured every 5-6 days. ThinCert® cell culture inserts were coated according to the manufacturer's instructions with rat-tail collagen type-I (10 µg or 50 µg/1.13 cm^2 insert) prior to seeding. Collagen stock solution (3 mg/mL) was diluted with sterile water to generate a 1.5 mg/mL intermediate solution which was then further diluted with aqueous ethanol (50% v/v) to give a working solution of 0.1 mg or 0.5 mg/mL. 100 µL of the working collagen solution was pipetted onto the insert membrane and allowed to air dry in the cell culture hood for 4 h. RPMI 2650 cells were seeded at different densities ($1x10^5$, $1.5x10^5$, $2x10^5$, $3.5x10^5$ and $5x10^5$ cells/1.13 cm^2 insert), and the cultures kept immersed in culture medium for 48 h (37 °C, 95% air humidity, 5% CO_2) with 800 µL and 200 µL in the basal and apical compartments, respectively. Air-liquid interface (ALI) cell cultures were established by removing the apical medium and maintaining the cells for 14 days, replacing the basal medium every 2-3 days to allow cell differentiation in terms of tight junction formation and mucus production prior to permeability studies as previously described.[1, 2]

S1.5. Transepithelial electrical resistance (TEER).

Using an epithelial voltohmmeter (EVOM, World Precision Instruments, USA), TEER values were monitored every 2-3 days during cell growth and differentiation to evaluate the tightness of the epithelial barrier and its validity for use in transport studies. Before each TEER measurement, 500 µL medium was introduced to the apical compartment and allowed to equilibrate in the

incubator (30 min, 37 °C, 5% CO_2). TEER values were corrected by subtracting values obtained from cell-free inserts and multiplying by the membrane surface area (1.13 cm^2).

S1.6. Mucus production.

Mucus secretion from RPMI 2650 cells seeded at different densities was visualized after 14 days of culture using Alcian blue glycoprotein staining as previously described.[3] In brief, culture inserts were washed with 300 µL of prewarmed PBS and subsequently fixed with 4% v/v paraformaldehyde (PFA) in PBS for 20 min at 20 °C. The inserts were then washed twice with PBS before adding 1% w/v Alcian blue solution (in 3% v/v glacial acetic acid, pH 2.5) to the apical compartment for 15 min at room temperature. The cells were then washed three times with 500 µL PBS and the inserts allowed to air dry for 3 h. The ThinCert® membrane was cut from the insert and mounted onto a glass microscope slide with Entellan™ new mounting medium, sealed and cooled to 4 °C before imaging using an inverted light microscope (Leica DMI 4000B, Leica Microsystems, Germany). Semi-quantitative analysis was performed using Fiji software[4] by measuring Red-Green-Blue (RGB) for each image and the calculating the RGB (blue) ratio using equation (S1), where b is blue, g is green, and r is red :

$$RGB_b\ ratio = \frac{RGB_b}{RGB_b + RGB_g + RGB_r} \qquad (S1)$$

S1.7. Tight junction visualization.

Tight junction proteins zonula occludens-1 (ZO-1) and E-cadherin were visualized by immunocytochemistry following 14 days of ALI culture as described by Ong et al.[3] Cells were rinsed three times with PBS then fixed with 4% v/v PFA for 10 min at room temperature. The cells were then permeabilized and blocked by incubating with Triton X-100 (0.2% w/v) for 10 min followed by a blocking buffer containing Triton X-100 (0.1% w/v) and bovine serum albumin (BSA;

1% w/v) in PBS for 60 min at 20 °C. Cells were then washed with PBS, incubated with 50 mM ammonium acetate in PBS for 10 min and washed once more with PBS. Cells were then incubated for 16 h at 37 °C with primary antibodies, either rabbit anti-zonula occludens-1 (D7D12, Cell Signalling Technology; 1:500) or rabbit anti-human E-cadherin (24E10, Cell Signalling Technology; 1:200) in blocking buffer. The cells were then washed three times with PBS and incubated for 1 h at 37 °C with a secondary antibody, goat anti-rabbit IgG labelled with Alexa fluor 488 (Invitrogen™; 1:100) in PBS. The samples were then rinsed three times with 500 µL PBS. The ThinCert® insert membrane was then detached from its plastic support, placed onto a glass microscope slide with cells facing up, and mounted with a mounting medium containing 4′,6-diamidino-2-phenylindone (DAPI) for nuclei staining (SlowFade™ Diamond Antifade Mountant with DAPI, Molecular Probes™), and stored at 4 °C prior to imaging with a confocal laser scanning microscope (LSM 880, ZEISS, Germany).

S1.8. Cell compatibility with FITC-PLGA NPs.

To assess the compatibility of FITC-PLGA nanoparticles with RPMI 2650 epithelial cells, the cells were exposed to aqueous NP suspensions, which were prepared using a previously reported protocol.[5] Briefly, an aqueous suspension of NPs (1% w/v) was diluted with culture medium to give three NP concentrations (0.05%, 0.1% and 0.2% w/v), which were maintained for 24 h at 25 °C. Cells were seeded in 96 well plates at 5x10^3 cells/well and cultured for 96 hours, replacing the culture medium after 48 h. For NP compatibility studies, the culture medium was removed from the cells, replaced with a 100 µL aliquot of NP suspension and the cells incubated with the FITC-PLGA NPs for 4 h or 48 h. The medium was then replaced with 200 µL of fresh, prewarmed medium containing 10% v/v of stock resazurin solution in PBS (0.15 mg/mL). Cells were incubated with the resazurin solution (2 h, 37 °C, 5% CO_2) and a 100 µL sample from each well then transferred into a fresh 96-well plate. The fluorescence of each well was measured at excitation

(λ_{ex}) and emission (λ_{em}) wavelengths of 540 nm and 590 nm, respectively using a FLUOstar Omega microplate reader (BMG Labtech, Germany). The measurements from NP-treated cells were normalized against untreated cells and, additionally, compared with a negative control of non-viable cells treated with Triton X-100 (0.2% w/v, 15 min).

S1.9. Epithelial permeability prior to aerosol exposure.

Two hydrophilic markers with different molecular weights were used to evaluate barrier formation and apical to basal permeability (A-B direction) of the ALI RPMI 2650 model following 14 days of culture. Flu-Na and FITC-Dextran (both 10 µg/mL) were dissolved separately in a buffer solution of HEPES (1% v/v) in HBSS, and 250 µL of marker solution then introduced into the apical chamber, with buffer alone (1 mL) added to the basal chamber. The cells were then incubated at 37 °C for 4 h with 100 µL samples withdrawn from the basal chamber at 30 min intervals and replaced with fresh buffer. Fluorescence readings were recorded using a CLARIOstar fluorescence microplate reader (BMG Labtech, Germany) at λ_{ex} 490 nm and λ_{em} 520 nm and the marker concentrations in the basal chamber determined using a calibration curve of the marker fluorescence signal against known concentrations. The cumulative mass of the fluorescent tracer transported across the barrier was determined by calculating the apparent permeability P_{app} coefficient (cm/s) using equation (S2):

$$P_{app}= \left(\frac{V}{C_0 \times A}\right) \times \left(\frac{dC}{dt}\right) \qquad \text{(S2)}$$

where V is the volume of the receiver chamber (cm^3), C_0 is the initial concentration of the fluorescent marker (µg/mL), A is the surface area of the insert (cm^2), and dC/dt is the rate of change of mass of the marker in the receiver chamber (µg/mL/s), in other words, the slope of the regression line obtained by plotting the cumulative mass of the permeated substance collected in the acceptor chamber against time, considering only the linear part of the graph.

S1.10. Cell uptake of FITC-PLGA nanoparticles.

To confirm that FITC-PLGA NPs were taken up by the cells and to monitor intracellular particle trafficking, confocal microscopy was performed.[5] NP suspensions were prepared at 0.05% and 0.1% w/v as described above (Section S1.8) and used following 2 h equilibration in cell culture medium. RPMI 2650 cells seeded in 35 mm glass-bottomed dishes (No. 1.5 glass coverslip; IBIDI, Germany) at a density of $3.5x10^6$ cells/dish and incubated for 4 days at 37 °C and 5% CO_2. Cell culture medium was replaced with NP suspensions, and the dishes were then incubated for 4 h or 24 h at 37 °C. The medium was removed, the cells were washed three times with HBSS (1 mL), and then kept immersed in HBSS for observation by confocal microscopy (LSM 880, ZEISS, Germany). A negative control of untreated cells was considered as the baseline.

For a quantitative analysis of the cellular uptake of FITC-PLGA NPs, flow cytometry was performed (FACSCanto, BD, US). Cells were seeded in a 12-well plate at $1x10^6$ cells/well and incubated for 3 days at 37 °C and 5% CO_2. The culture medium was then placed with the NP suspension (0.1% w/v in culture medium) and the cells incubated at 37 °C for 4 h or 24 h. The medium was removed, and the cells were gently washed twice with fresh prewarmed medium. The cells were then harvested and finally resuspended in 200 µL fresh medium for flow cytometry analysis (λ_{ex} 488 nm, λ_{em} 530 nm). For each cell suspension, 20,000 events were measured, and the data were analysed using the software FCS Express (v 7.14.0020).

S1.11. Statistical analysis.

Where applicable, data were analysed using unpaired two-tailed student's t-test or one-way ANOVA followed by post-hoc Tukey's HSD analysis using GraphPad Prism 9.4.1 software. All data were expressed as mean ± standard deviation (SD). A value of $P < 0.05$ was considered to be statistically significant.

S2. Results

S2.1. Emitted FITC-PLGA NP dose

The emitted dose of NPs from a pMDI device was examined by firing six sets of five shots into a glass vial and determining the mass of particles deposited. Figure S1 shows the emitted doses from shots 4 to 33, with the dose recovered from the glass vial compared to that recovered from the actuator of the pMDI. The group from shot 4 to 8 had the highest total dose while, beyond that, the doses were broadly comparable with a mean dose across all groups of 232±76 µg. Some variation was clearly apparent, but this is to be expected due to heterogeneous particle mass fraction distributed in the aerosol droplets.[6,7]

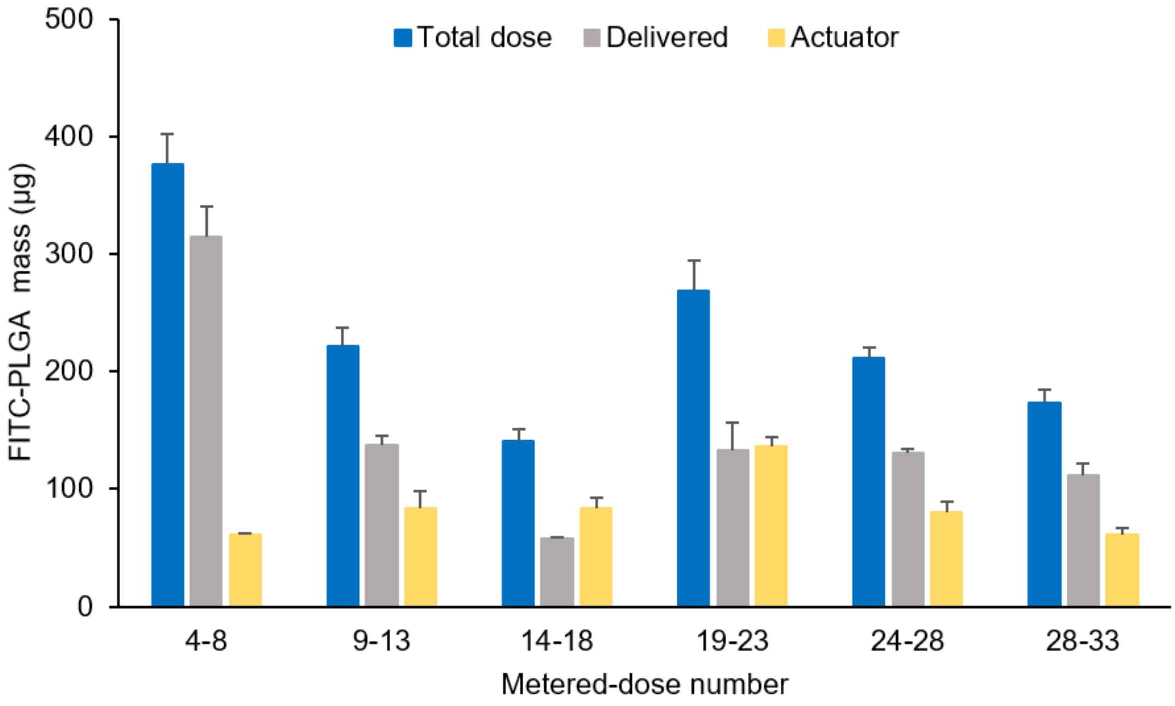

Figure S1. Delivered dose uniformity test (mean ± SD, n = 5) for six sets of five shots of NP-based pMDI represented by the recovered FITC-PLGA mass.

S2.2. TEER measurements for a ThinCert® ALI RPMI 2650 model.

The effect of five different cell seeding densities and two collagen coating concentrations was examined for RPMI 2650 cells cultured on ThinCert® inserts under ALI conditions. Figure S2B shows the developing TEER values with respect to time over 18 days. For all seeding densities,

a regular TEER increase was reported, with the highest value around 102 Ω.cm^2 achieved at day 10 for cells seeded on an insert coated with 50 µg collagen and seeded at a density of 3.5x10^5 cells per insert. This correlated with the formation of a homogenous and confluent multilayer of cells (Figure S2A). Higher seeding densities with lower collagen coating densities led to cell detachment from the insert membrane within 7 days, whereas lower densities experienced a sudden and steep TEER decrease on day 12 post-seeding. No plateau phase of consistent TEER values was observed for the applied conditions, except for cells seeded at a density of 1.0x10^5 cells per insert, which maintained a maximum TEER value of ~60 Ω.cm^2 between 8- and 12-days post-seeding.

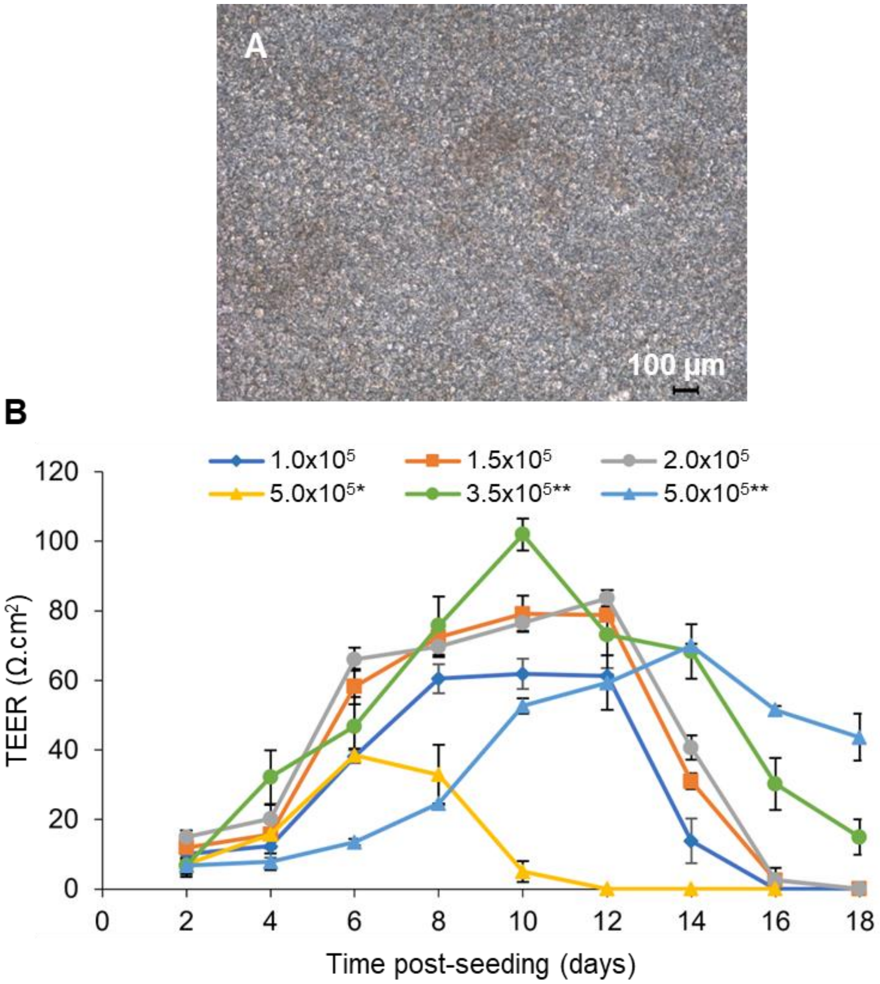

Figure S2. A) Light microscope image for the confluent ALI RPMI 2650 cell layers grown on collagen-coated ThinCert® inserts. B) TEER (mean ± SD, n = 6) for RPMI 2650 cells seeded on a coated ThinCert® membranes (1.13 cm^2) coated with 10 µg collagen and cultured under ALI after 48 h. *Discontinued at day 10. **The ThinCert® membrane was pre-coated with 50 µg collagen.

S2.3. Mucus production.

Mucus production by RPMI 2650 cells was determined by Alcian blue staining of mucin proteins. Cells were seeded at various densities and maintained for 14 days under ALI culture before being incubated with Alcian blue solution. Images of the stained cells were analysed by colour density mapping using the Fiji Color Inspector 3D (v2.5) plugin and the level of blue staining was quantified. Using all colour and median cut algorithms (a function used for colour quantization by sorting the data into the best representative colour subsets), the density of colour mapping was created within a cubic space in which the three axes x, y, and z represent red, green, and blue, respectively. Each pixel from the images was assigned a colour value which was then allocated a coordinate in the cubic space. The images were also analysed by calculating the average blue ratio (RGB_b) of three images for each growth condition. Figure S3A shows microscopic images of the cells at different seeding densities following Alcian blue staining after 14 days of ALI culture, with the density of colour mapping with all colours (Figure S3B) and median cut algorithm (Figure S3C). It can be seen that mucus was produced and distributed over the entire cell layer with a uniform and intense colour covering all the areas for the studied densities with darker blue spots observed for seeding densities $\geq 2.0 \times 10^5$ cells per insert.

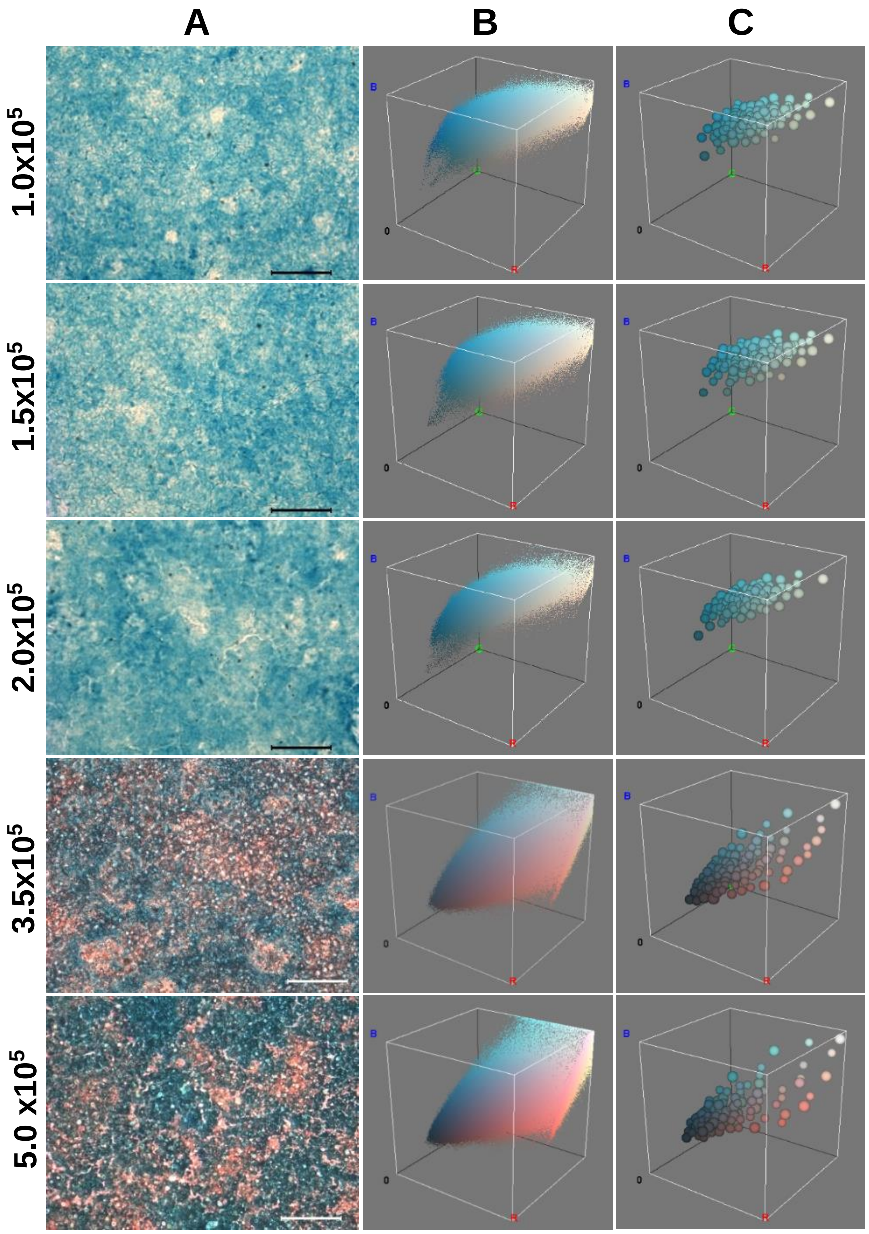

Figure S3. Analysis of mucin protein labelling with Alcian blue for different RPMI 2650 cell seeding densities grown on ThinCert® inserts under ALI for 14 days. A) Optical microscopic images. B) Colour density mapping for all colours, and C) density mapping after modification using the median cut algorithm. The scale bars are 100 µm.

Further relative quantification of the mucus secretion was determined by the RGB_b ratio analysis as shown in Figure S4. Under all seeding conditions no significant differences were noticed after 2 weeks of ALI culture ($P > 0.05$). These observations, along with the TEER data, confirm that 14

days is sufficient for RPMI 2650 cells cultured under these conditions to proliferate with and establish a homogenous mucus-secreting epithelial barrier.

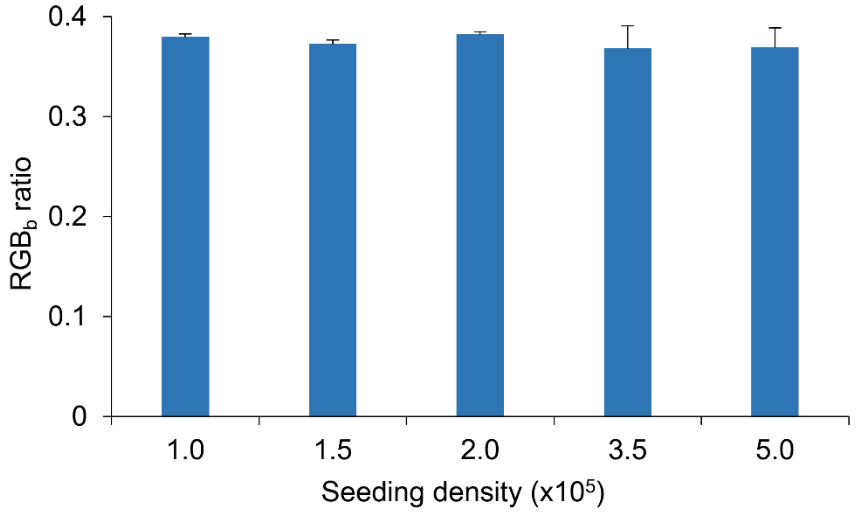

Figure S4. The mean RGB$_b$ ratio (± SD, n = 3) following mucus staining of 14-day ALI RPMI 2650 cultures with Alcian blue.

S2.4. Tight junction protein visualization.

The integrity and functionality of epithelial barriers are directly correlated with the formation of tight junctions (TJs) between epithelial cells. The expression of essential TJ proteins ZO-1 and E-cadherin, and therefore the functional integrity of the RPMI 2650 cell model, was examined by immunocytochemistry and subsequent visualization using confocal microscopy as shown in Figure S5. ALI RPMI 2650 cultures at 14 days expressed both proteins at the periphery of the cells in agreement with previous studies for RPMI 2650 cultured under ALI conditions.[1, 2, 8]

S2.5. Cell compatibility with the PLGA NP formulation.

The possibility of any gross adverse effects of the FITC-PLGA NP formulation on the RPMI 2640 cells was assessed using the resazurin bioassay, where three incremental working concentrations of NPs (0.05, 0.1, and 0.2% w/v) were tested following direct dilution with growth medium. Figure S6 shows the metabolic activity of the treated cells compared to the control following 4 h and 48

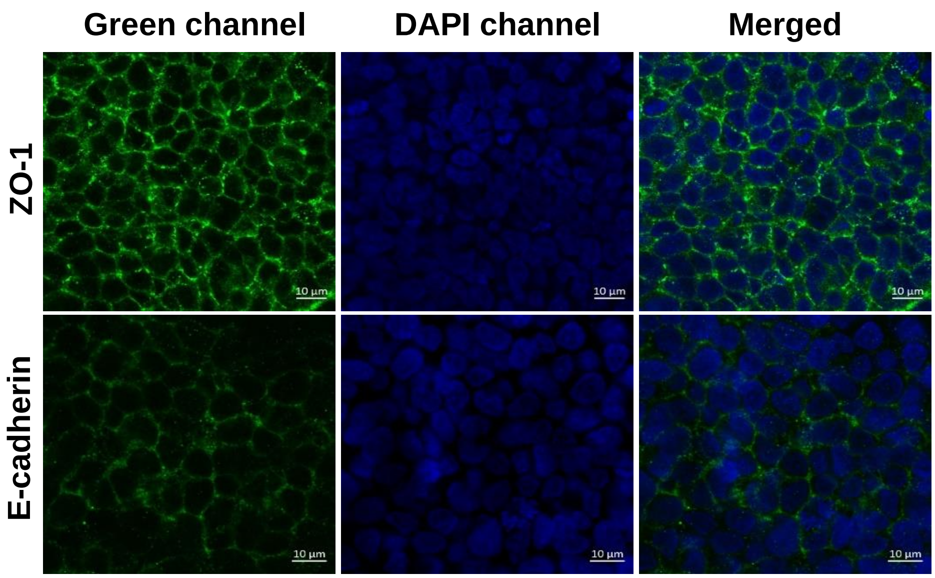

Figure S5. Confocal laser scanning microscopy images for 14-day ALI RPMI 2650 cultures grown on ThinCert® membranes, showing the expression of TJ proteins ZO-1 and E-cadherin.

h incubation with the NP formulations. The aqueous NP suspension demonstrated excellent compatibility with RPMI 2650 cells with none of the three concentrations affecting the metabolic activity of the cells at either timepoint. Ensuring cell compatibility was crucial for assessing the concentration thresholds for permeability assays. In addition, in this study, the formulation mass

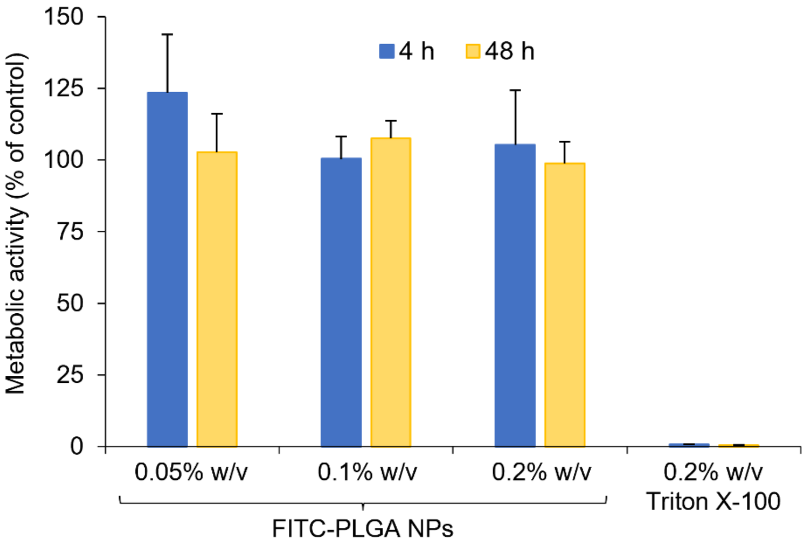

Figure S6. RPMI 2650 cell metabolic activity (mean ± SD, n = 6) when exposed to FITC-PLGA NPs at different concentrations for 4 h and 48 h using the resazurin assay. Data are normalized against control, untreated cells.

loading into the device and the likely total number of administered doses should be within the range of the non-harmful concentrations observed here.

S2.6. Epithelial permeability prior to aerosol exposure.

As stated above, RPMI 2650 cells exhibited TEER values below 100 Ω.cm^2, forming a loose multi-layered epithelium. To further investigate the barrier function of RPMI 2650 cultures, Flu-Na and FITC-dextran were used as fluorescent tracers to determine upper-to-lower compartment (apical-basal) permeation. The RPMI 2650 nasal epithelial barrier was established using cells seeded at 3.5x10^5 cells per membrane and grown under ALI conditions for 14 days. Many studies have reported that RPMI 2650 epithelial cells have improved differentiation, forming a tighter barrier, when they are in direct contact with the ambient air rather than immersed in cell culture medium. A direct comparison of ALI and liquid-liquid interface was beyond the scope of this study but has been previously investigated.[1, 5, 9]

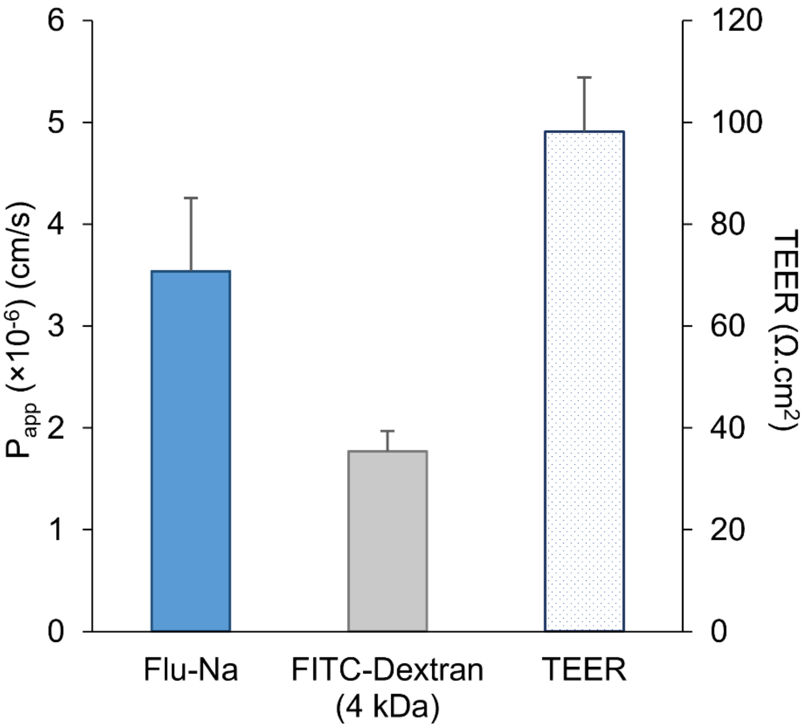

Figure S7. The epithelial permeability (mean ± SD, n = 3) of Flu-Na and 4 kDa FITC-dextran after 4 h incubation with 14-day RPMI 2650 ALI cultures and the TEER of the cells (mean ± SD, n = 9).

Figure S7 shows that the ALI RPMI 2650 cultures in this study presented a P_{app} value of 3.5x10^{-6} cm/s for Flu-Na. This value corresponds well with the permeation coefficient of 3.12x10^{-6} cm/s for excised human nasal mucosa reported by Wengst and Reichl, although the authors also stated high interindividual variabilities of 3.12 ± 1.99x10^{-6} cm/s.[1] For FITC-dextran (4 kDa) the

permeation coefficient of 1.7x10^{-6} cm/s was around 2-fold lower than that of Flu-Na (376.3 Da) due to its higher molecular weight, and is also lower than the previously reported value of 2.48x10^{-6} cm/s for FITC-dextran of equivalent molecular weight,[1] suggesting an improved barrier function in this study.

S2.7. Uptake of FITC-PLGA NPs by RPMI 2650 cells.

The relationship between FITC-PLGA NP concentrations and their uptake efficiency into the nasal epithelial cells was studied over a 24 h period. The visualization of individual NPs within the cells was not straightforward using confocal microscopy, and only aggregates of particles could be observed. As such, the green fluorescence intensity of FITC, as an indicator of uptake of the formulation, was screened under the confocal microscope after 4 h, and 24 h exposure of cells to FITC-PLGA NP suspensions at 0.05% and 0.1% w/v. Figure S8 shows the fluorescent confocal micrographs for the two time points. Most studies have reported high cellular uptake efficiency and viability for polymeric NPs as NTBDD systems due to their suitable sizes and biocompatibility. In this study, the particles were clearly taken up by the cells after 4 h exposure for both

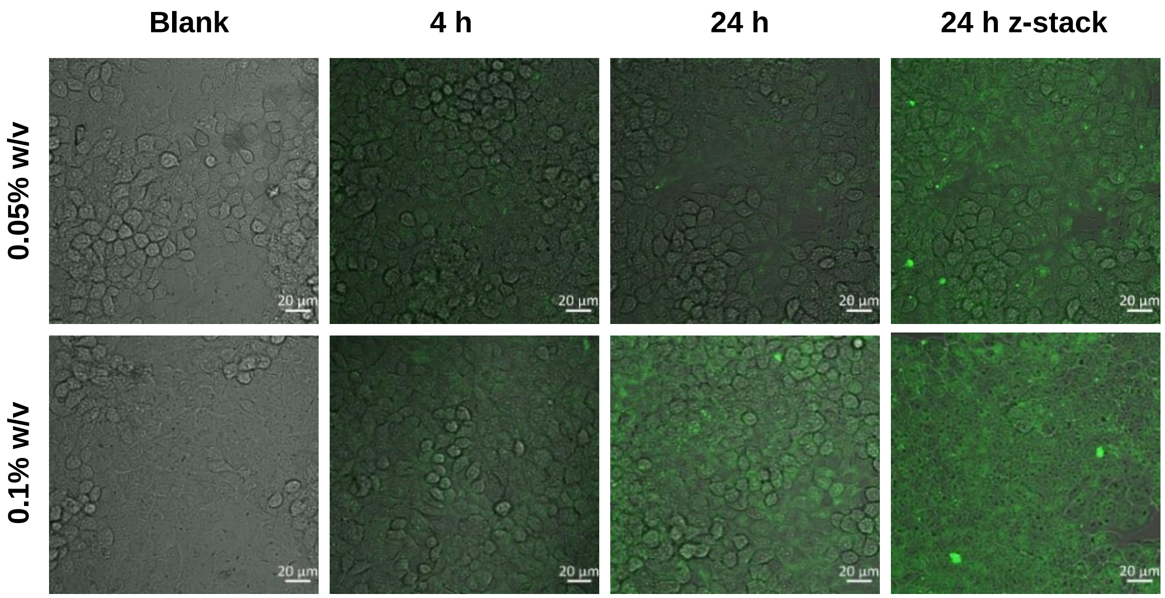

Figure S8. Merged brightfield and green fluorescence confocal micrographs of RPMI 2650 nasal cells exposed to 0.05% or 0.1% w/v aqueous suspensions of FITC-PLGA NPs for 4 or 24 h at 37 °C. The blank shows the cell layers with no NP exposure.

concentrations, and the extent of the uptake was significantly higher following 24 h exposure to the formulation, which was also confirmed using Z-stack analysis.

Although both concentrations examined in this study followed a similar uptake pattern in terms of time dependency, at 4 h treatment fluorescent intensities were comparable between the low (0.05%) and the high (0.1%) NP working concentrations, whereas there were distinct differences after 24 h (Figure S8). This might be due to the formation of some aggregates and larger particles when the NPs were introduced to the complex cell culture medium, which could have had an impact on their translocation. The likely formed aggregates in this study were at the expense of smaller particles and might have been excluded from some size-dependent cellular entry pathways. As such, the translocation might be dominantly paracellular across the leaky cell layers.[10, 11]

The extent of NP uptake into RPMI 2650 cells was further quantified by flow cytometry analysis. Cells were treated with a 0.1% w/v NP suspension for 4 h or 24 h, harvested and resuspended in fresh, prewarmed medium for the analysis. The quantitative output was the percentage of the cells stained with the NPs following the washing steps. The statistical significance for the FITC median fluorescent intensity between the different exposure times was tested by Student's *t*-test or one-way analysis of variance ANOVA. Regardless of the incubation time at 37 °C, >99% of the cells were found to have green fluorescence. As shown in Figure S9, significant differences ($P < 0.0005$) were observed between the control and FITC-PLGA NP groups as well as between the two incubation times. An approximate 2-fold increase in the fluorescent intensity was achieved after 24 h exposure compared to the 4 h time point.

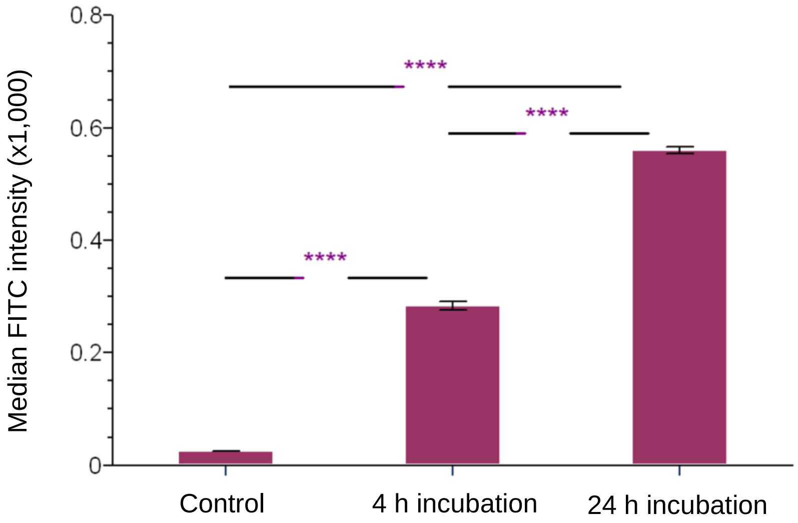

Figure S9. Flow cytometry analysis of RPMI 2650 cells treated with 0.1% w/v FITC-PLGA NPs for 4 and 24 h at 37 °C (mean ± SD, n = 3; **** P <0.005).

References

(1) Wengst, A.; Reichl, S. RPMI 2650 epithelial model and three-dimensional reconstructed human nasal mucosa as in vitro models for nasal permeation studies. *Eur J Pharm Biopharm* **2010**, *74* (2), 290-297. DOI: 10.1016/j.ejpb.2009.08.008.

(2) Mercier, C.; Hodin, S.; He, Z.; Perek, N.; Delavenne, X. Pharmacological Characterization of the RPMI 2650 Model as a Relevant Tool for Assessing the Permeability of Intranasal Drugs. *Mol Pharm* **2018**, *15* (6), 2246-2256. DOI: 10.1021/acs.molpharmaceut.8b00087.

(3) Ong, H. X.; Jackson, C. L.; Cole, J. L.; Lackie, P. M.; Traini, D.; Young, P. M.; Lucas, J.; Conway, J. Primary Air-Liquid Interface Culture of Nasal Epithelium for Nasal Drug Delivery. *Mol Pharm* **2016**, *13* (7), 2242-2252. DOI: 10.1021/acs.molpharmaceut.5b00852.

(4) Schindelin, J.; Arganda-Carreras, I.; Frise, E.; Kaynig, V.; Longair, M.; Pietzsch, T.; Preibisch, S.; Rueden, C.; Saalfeld, S.; Schmid, B.; et al. Fiji: an open-source platform for biological-image analysis. *Nat Methods* **2012**, *9* (7), 676-682. DOI: 10.1038/nmeth.2019.

(5) Schlachet, I.; Sosnik, A. Mixed Mucoadhesive Amphiphilic Polymeric Nanoparticles Cross a Model of Nasal Septum Epithelium in Vitro. *ACS Appl Mater Interfaces* **2019**, *11* (24), 21360-21371. DOI: 10.1021/acsami.9b04766.

(6) Chierici, V.; Cavalieri, L.; Piraino, A.; Paleari, D.; Quarta, E.; Sonvico, F.; Melani, A. S.; Buttini, F. Consequences of not-shaking and shake-fire delays on the emitted dose of some

commercial solution and suspension pressurized metered dose inhalers. *Expert Opin Drug Deliv* **2020**, *17* (7), 1025-1039. DOI: 10.1080/17425247.2020.1767066.

(7) Stein, S. W. Estimating the number of droplets and drug particles emitted from MDIs. *AAPS PharmSciTech* **2008**, *9* (1), 112-115. DOI: 10.1208/s12249-007-9006-8.(8) Mercier, C.; Perek, N.; Delavenne, X. Is RPMI 2650 a Suitable In Vitro Nasal Model for Drug Transport Studies? *Eur J Drug Metab Pharmacokinet* **2018**, *43* (1), 13-24. DOI: 10.1007/s13318-017-0426-x.

(9) Gerber, W.; Svitina, H.; Steyn, D.; Peterson, B.; Kotze, A.; Weldon, C.; Hamman, J. H. Comparison of RPMI 2650 cell layers and excised sheep nasal epithelial tissues in terms of nasal drug delivery and immunocytochemistry properties. *J Pharmacol Toxicol Methods* **2022**, *113*, 107131. DOI: 10.1016/j.vascn.2021.107131.

(10) Bejgum, B. C.; Donovan, M. D. Uptake and Transport of Ultrafine Nanoparticles (Quantum Dots) in the Nasal Mucosa. *Mol Pharm* **2021**, *18* (1), 429-440. DOI: 10.1021/acs.molpharmaceut.0c01074.

(11) Chen, N. Size and surface properties determining nanoparticle uptake and transport in the nasal mucosa. (PhD thesis, University of Iowa, Iowa, USA), **2013**. DOI: 10.17077/etd.8gfca4jk.
